# Phytochemical Characterization of Freeze-Dried *Stenocereus stellatus* Seeds and Their Antihyperglycemic Activity in Experimental Prediabetes and Type 2 Diabetes

**DOI:** 10.3390/molecules31172964

**Published:** 2026-08-25

**Authors:** Víctor Hugo Oidor-Chan, Fernando Díaz de León-Sánchez, Leonardo del Valle-Mondragón, Raúl Martínez-Memije, Juan Carlos Torres-Narváez, Francisco Cruz-Sosa, Mariana Patlán, Manuel Miranda, Melani León-Martínez, Victoria López-Olmos, Luz Ibarra-Lara, Vicente Castrejón-Téllez

**Affiliations:** 1 Department of Biotechnology, Metropolitan Autonomous University, Iztapalapa Campus, Av. Ferrocarril de San Rafael Atlixco 186, Col. Leyes de Reforma 1ª. Sección, Alcaldía Iztapalapa, Mexico City 09310, Mexico; victorhugooidor@xanum.uam.mx (V.H.O.-C.); cuhp@xanum.uam.mx (F.C.-S.); 2 Laboratory of Post-Harvest of Plant Genetic Resources and Natural Products, Department of Health Sciences, Metropolitan Autonomous University, Iztapalapa Campus, Av. Ferrocarril de San Rafael Atlixco 186, Col. Leyes de Reforma 1ª. Sección, Alcaldía Iztapalapa, Mexico City 09310, Mexico; fdls@xanum.uam.mx; 3 Department of Pharmacology, Instituto Nacional de Cardiología “Ignacio Chávez”, Juan Badiano No. 1, Col. Sección XVI, Tlalpan, Mexico City 14080, Mexico; leonardo.delvalle@cardiologia.org.mx (L.d.V.-M.); juancarlostn63@hotmail.com (J.C.T.-N.); 4 Department of Electromechanical Instrumentation, National Institute of Cardiology Ignacio Chavez, Mexico City 14080, Mexico; raul.martinez@cardiologia.org.mx; 5 Subdirection of Basic and Technological Research, Instituto Nacional de Cardiología “Ignacio Chávez”, Mexico City 14080, Mexico; mariana.patlan@cardiologia.org.mx; 6 Department of Biological Sciences, University of Texas at El Paso, Bioscience Building, 500 West University Avenue, El Paso, TX 79968, USA; mmiranda3@utep.edu; 7 Department of Physiology, Instituto Nacional de Cardiología “Ignacio Chávez”, Juan Badiano No. 1, Col. Sección XVI, Tlalpan, Mexico City 14080, Mexico; melanileonmtz@gmail.com (M.L.-M.); victoria.lopez@cardiologia.org.mx (V.L.-O.)

**Keywords:** *Stenocereus stellatus*, freeze-dried seeds, phytochemicals profiling, prediabetes, type 2 diabetes, glucose tolerance, polyphenols, insulin responsiveness

## Abstract

Background: Prediabetes and type 2 diabetes (T2D) are characterized by progressive impairment of glucose homeostasis. This study aimed to expand the phytochemical profile of freeze-dried *Stenocereus stellatus* (red tunillo) seeds and evaluate their antihyperglycemic activity in experimental models of prediabetes (PDB) and T2D. Results: Phytochemical screening and UPLC-QTOF-MSE revealed a chemically diverse profile. Six chromatographic features were putatively annotated on the basis of accurate mass, elemental composition, isotopic pattern, and high-energy data; the confidence of these annotations varied among features because authentic standards were not analyzed. Fourteen-day oral administration of freeze-dried seeds reduced blood glucose and improved glucose tolerance in prediabetic rats. In T2D rats, treatment reduced hyperglycemia and enhanced the glucose-lowering response to exogenous insulin, although the overall glucose excursion during the OGTT was not significantly improved. Methods: Female rats with PDB and male rats with T2D were fed freeze-dried *S. stellatus* seeds at 100 and 200 mg kg^−1^, respectively, for 14 consecutive days. Conclusions: Freeze-dried *S. stellatus* seeds showed antihyperglycemic activity in both experimental models. Further quantitative, mechanistic, and long-term studies are required to define the contribution of individual metabolites and assess translational relevance.

## 1. Introduction

Prediabetes and type 2 diabetes (T2D) represent major global health challenges because of their increasing prevalence and association with cardiovascular, renal, neurological, and ocular complications. Persistent hyperglycemia is a central metabolic alteration in both conditions and contributes to progressive tissue dysfunction. Current pharmacological therapies are effective for glycemic control; however, approaches based on dietary and plant-derived bioactive compounds continue to be investigated as potential strategies to support metabolic health [1,2,3].

Plant-derived natural products have attracted considerable attention as potential sources of bioactive compounds with antihyperglycemic properties. Among these, polyphenols constitute one of the most extensively investigated classes because they regulate multiple molecular pathways involved in glucose metabolism, including AMP-activated protein kinase (AMPK), phosphatidylinositol-3 kinase/protein kinase B (PI3K/Akt), peroxisome proliferator-activated receptors (PPARs), and nuclear factor erythroid 2-related factor 2 (Nrf2). Through these mechanisms, polyphenols improve insulin sensitivity, preserve pancreatic β-cell function, reduce oxidative stress, and contribute to the maintenance of glucose homeostasis [3,4].

Botanical materials contain chemically diverse metabolites that may contribute to their biological properties. Therefore, phytochemical profiling is an important step for describing the chemical composition of natural preparations and identifying candidate constituents for subsequent investigation. Advances in ultra-performance liquid chromatography coupled with high-resolution mass spectrometry (UPLC-QTOF-MS) have improved the analysis of complex botanical matrices by providing accurate-mass and fragmentation information that supports metabolite annotation [4,5].

Mexico is one of the principal centers of diversification of the family Cactaceae, with approximately 550–900 species, nearly 79% of which are endemic [6]. Among them, *Stenocereus stellatus* (red tunillo) is native to the Mixteca region of Oaxaca and Puebla and produces edible fruits traditionally consumed fresh or processed into beverages, jams, frozen desserts, and dehydrated products [7,8]. Previous studies have demonstrated that the fruits contain abundant phenolic compounds, betalains, and vitamin C, exhibiting remarkable antioxidant capacity and nutritional value [9,10].

Previous phytochemical studies of *S. stellatus* have focused mainly on the fruit pulp. Chromatographic analyses have shown that red *S. stellatus* fruits contain a complex betalain profile, including indicaxanthin, gophrenin I, phyllocatin, and related isomers. The phenolic fraction is also chemically diverse and includes hydroxycinnamoyl derivatives, flavonols, and flavanones. In addition, substantial amounts of total phenolic compounds, betacyanins, and betaxanthins have been reported in red *S. stellatus* fruit preparations. These findings support the presence of a chemically diverse group of secondary metabolites in this species. However, most previous studies have focused on the fruit pulp, whereas the phytochemical composition of the seeds remains much less characterized [5,8,9].

Approximately one-third of the edible fruit corresponds to the seeds, representing nearly 31% of the total biomass [11,12]. Despite their abundance, the phytochemical composition of red tunillo seeds has received considerably less attention than that of the pulp. Previous investigations have identified flavonoids such as quercetin and rutin [13], while experimental studies from our group demonstrated that freeze-dried preparations of tunillo pulp and seeds significantly improved glucose tolerance in healthy rats subjected to oral glucose overload [5,12]. More recently, freeze-dried seeds were shown to reverse experimental prediabetes through mechanisms associated with activation of the hepatic eNOS/Akt signaling pathway [5]. These findings suggest that tunillo seeds represent a promising source of bioactive phytochemicals capable of modulating glucose metabolism.

Previous studies from our group showed that freeze-dried preparations of *S. stellatus* pulp and seeds improved glucose tolerance in healthy rats subjected to an acute oral glucose challenge. More recently, freeze-dried seeds were shown to improve metabolic alterations in an experimental model of prediabetes through mechanisms associated with hepatic eNOS/Ak signaling. However, the phytochemical composition of the freeze-dried seed preparation remains incompletely characterized, and its antihyperglycemic activity in established T2D has not been evaluated.

Therefore, the present study was designed to extend our previous findings by providing a broader phytochemical characterization of freeze-dried *S. stellatus* seeds using UPLC-QTOF-MS and by evaluating their metabolic effects in experimental models of both prediabetes and T2D. Because the two models differed in sex and treatment dose, they were analyzed independently and were not intended for direct comparison of treatment efficacy between disease stages.

## 2. Results

### 2.1. Phytochemical Characterization of Freeze-Dried Stenocereus stellatus Seeds

Qualitative phytochemical analysis of the aqueous suspension (10%, *w*/*v*) of freeze-dried *S. stellatus* seeds confirmed the presence of phenolic compounds. All independent determinations produced the characteristic blue coloration following the Folin–Ciocalteu reaction, indicating the occurrence of reducing phenolic constituents (Total Phenolic Content) in the seed preparation.

**Figure 1:**
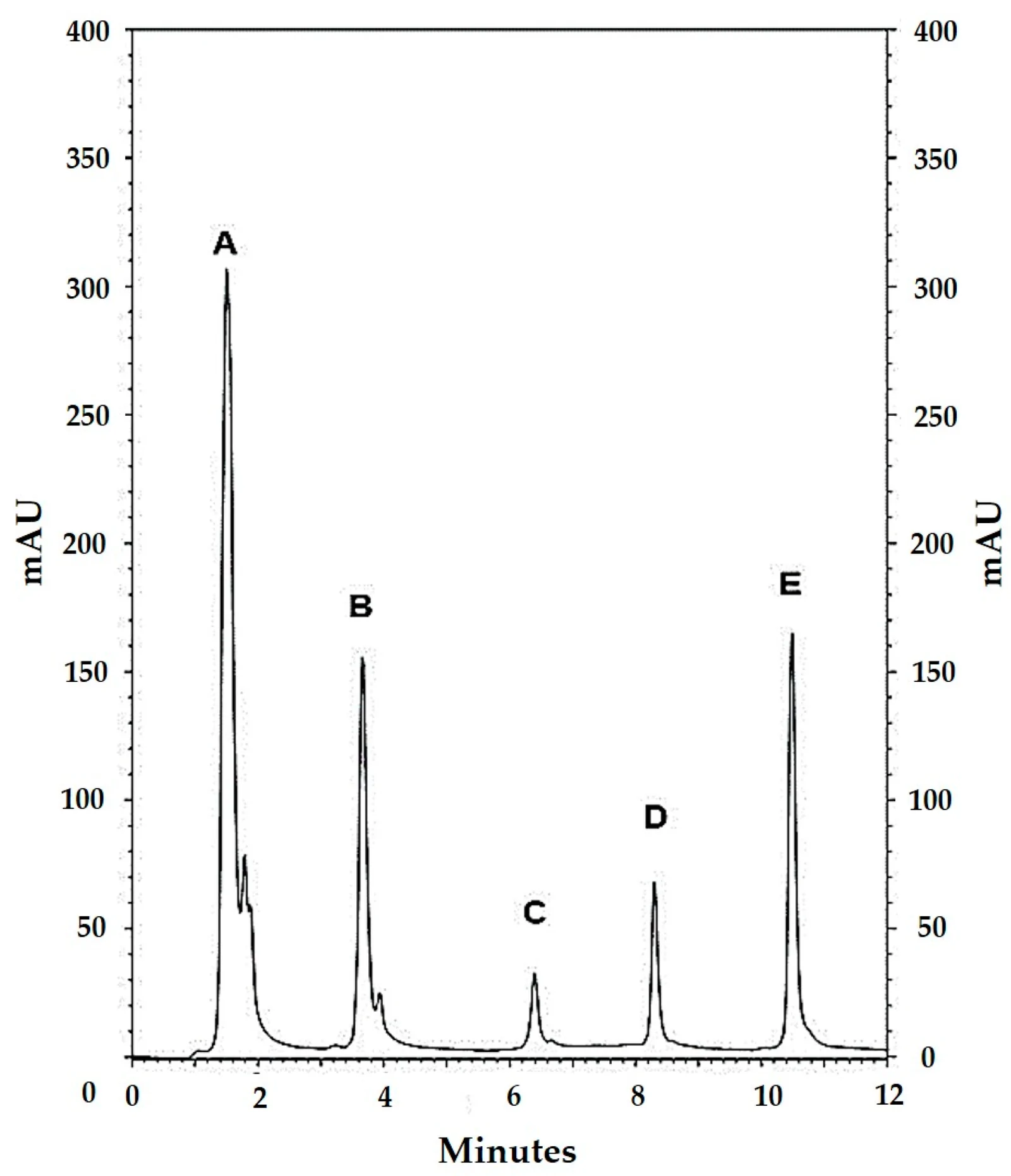
UPLC chromatogram of the 10% (*w*/*v*) aqueous preparation of freeze-dried red tunillo (*Stenocereus stellatus*) seeds. Five well-resolved peaks (A–E) are observed.

UPLC analysis revealed a relatively simple chromatographic profile composed of five well-resolved major peaks, designated A–E (Figure 1). The high chromatographic resolution enabled individual collection of each fraction for subsequent chemical characterization.

To determine total phenolic content within the chromatographic profile, each fraction was analyzed individually using the Folin–Ciocalteu assay. Only fractions A and B yielded positive reactions, whereas fractions C, D, and E showed no detectable phenolic response. Quantification of the Folin–Ciocalteu-positive fractions demonstrated that fraction A contained a higher concentration of phenolic compounds than fraction B, expressed as gallic acid equivalents.

**Figure 2:**
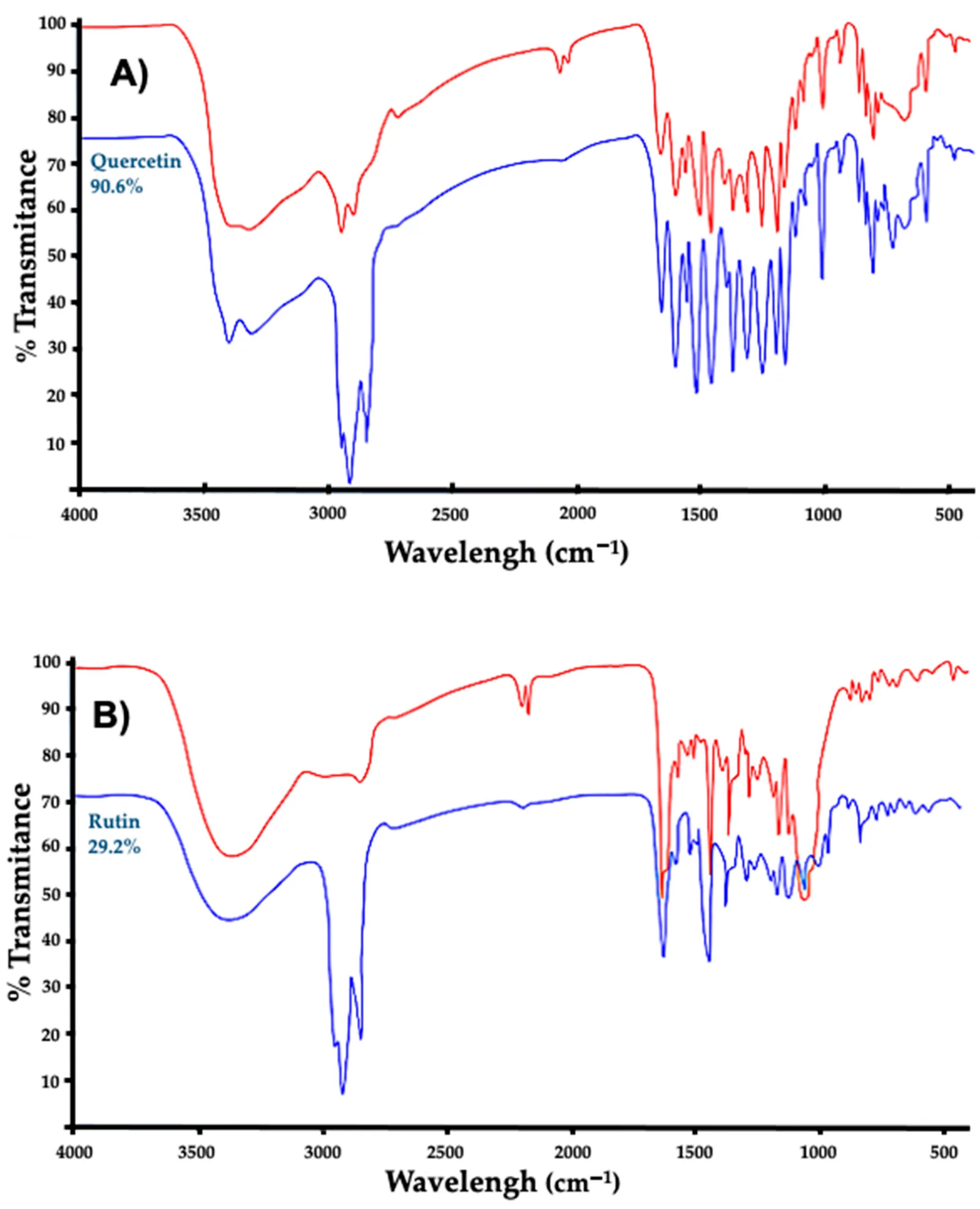
FT-IR spectra of (**A**) fraction A (blue line) compared with the quercetin reference spectrum from the instrument library (red line), showing 90.6% spectral similarity, and (**B**) fraction B (blue line) compared with the rutin reference spectrum from the instrument library (red line), showing 29.2% spectral similarity.

To obtain additional structural information, fractions A and B were analyzed by Fourier-transform infrared spectroscopy (FTIR). Although both fractions contained phenolic compounds, their infrared spectra demonstrated distinct molecular fingerprints, indicating that they corresponded to chemically different constituents. Spectral comparison against the instrument library showed that fraction A exhibited 90.6% similarity with quercetin, whereas fraction B showed 29.2% similarity with rutin (Figure 2). The retention times obtained by UPLC further supported their chromatographic separation, with fraction (A) eluting at 1.43 min and fraction (B) at 3.69 min.

Fractions C, D, and E were also concentrated for FTIR analysis. However, only fraction C yielded sufficient solid material for spectral evaluation, whereas fractions D and E formed viscous residues that could not be adequately purified for analysis. The infrared spectrum obtained from fraction C did not match any reference compound available in the spectral library.

Because betalains have been reported in several red- and purple-colored fruits belonging to the genus *Stenocereus*, fractions C–E were subsequently evaluated for the presence of betanin. Among these fractions, only fraction C produced the characteristic color change associated with betanin following reaction with antimony (III) chloride, whereas fractions D and E tested negative. These findings indicate that, in addition to phenolic compounds, freeze-dried *S. stellatus* seeds contain betalain-derived pigments that may contribute to their phytochemical complexity.

Qualitative phytochemical screening of the aqueous preparation of freeze-dried *S. stellatus* seeds showed a positive Folin–Ciocalteu reaction, supporting the presence of phenolic constituents. UPLC separation yielded five well-resolved major fractions (A–E). Fractions A and B showed a positive Folin–Ciocalteu reaction, whereas fractions C–E showed no detectable response. Quantitative analysis of the Folin–Ciocalteu-positive fractions showed a higher phenolic content in fraction A than in fraction B, expressed as gallic acid equivalents. In addition, fraction C showed a positive reaction for betanin, whereas fractions D and E were negative.

To extend the chemical characterization of the seed preparation, a 1% (*w*/*v*) aqueous suspension was analyzed by UPLC-QTOF-MSE. Six chromatographic features were annotated at different confidence levels on the basis of accurate precursor mass, elemental composition, isotopic pattern, and associated high-energy ions (Table 1). Because authentic reference standards were not analyzed, none of these assignments should be considered an unequivocal structural identification. Representative low- and high-energy spectra are provided in the Supplementary Materials.

Overall, the analyses showed that freeze-dried *S. stellatus* seeds contain phenolic constituents and other detectable phytochemical features. UPLC-QTOF-MSE expanded the chemical profile of the aqueous seed preparation, while also showing that the confidence of individual metabolite assignments varies and requires confirmation with authentic standards and targeted structural analysis.

### 2.2. Validation of the Experimental Models of Prediabetes and Type 2 Diabetes

Eight weeks after neonatal administration of streptozotocin (STZ), female and male Wistar rats developed distinct metabolic phenotypes consistent with experimental PDB and T2D, respectively.

Non-fasting blood glucose concentrations were significantly more elevated in both STZ-treated groups than in their corresponding controls. Female rats exhibited moderate hyperglycemia (165.13 ± 4.97 mg dL^−1^ vs. 122.33 ± 2.97 mg dL^−1^ in controls), whereas male rats developed a more pronounced hyperglycemic state (241.17 ± 22.07 mg dL^−1^ vs. 121.38 ± 0.89 mg dL^−1^ in controls), indicating a more severe metabolic impairment.

**Figure 3:**
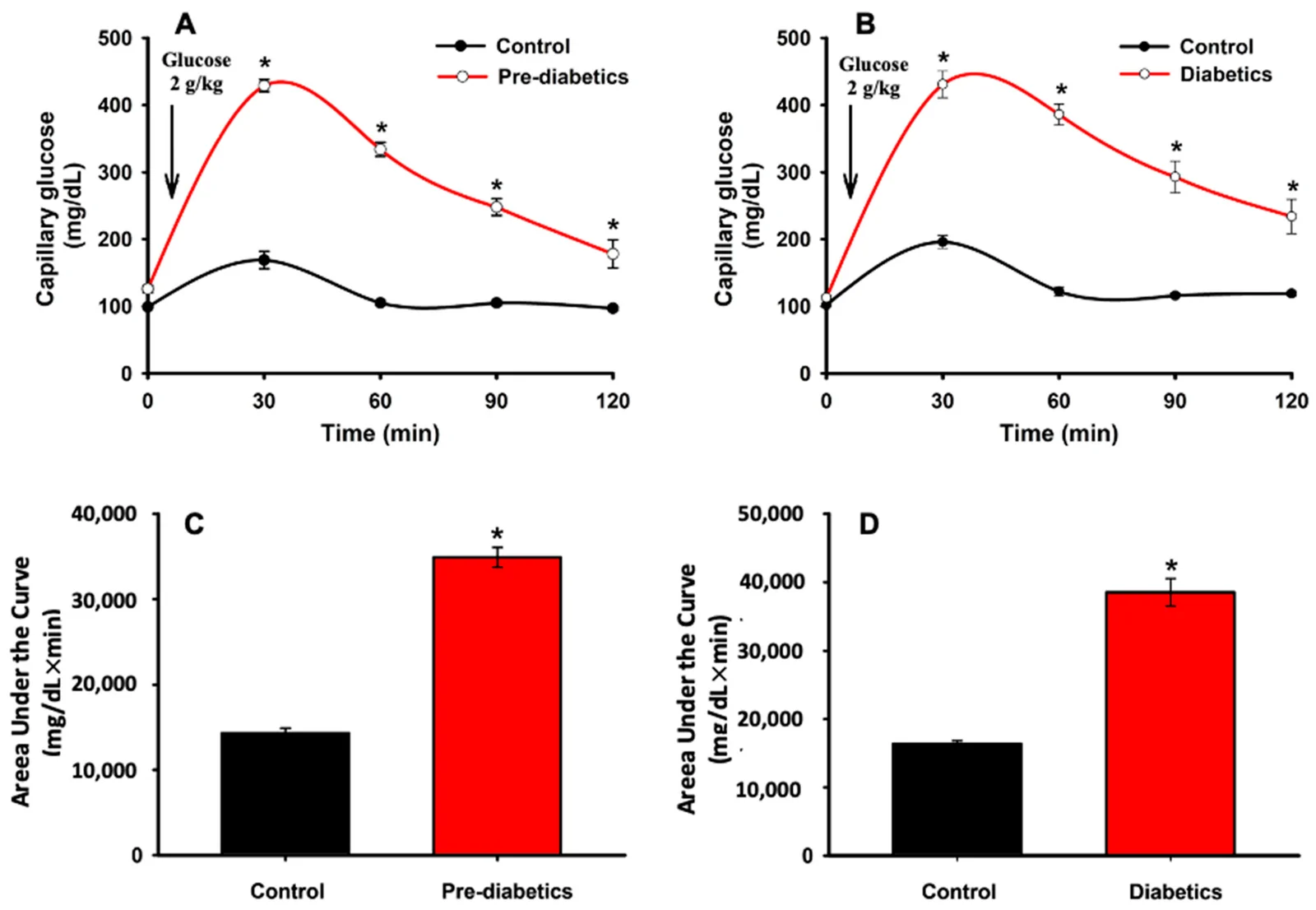
Validation of glucose intolerance in experimental prediabetes and type 2 diabetes (T2D). Oral glucose tolerance tests (OGTTs) performed eight weeks after neonatal streptozotocin administration in (**A**) female PDB rats and (**B**) male rats with T2D. Corresponding areas under the curve (AUCs) are shown in (**C**) female PDB rats and (**D**) male T2D rats. Data are presented as the mean ± SEM (*n* = 6 per group). * = *p* < 0.05 versus the corresponding control group (Two-way repeated-measures ANOVA followed by the student–Newman–Keuls post hoc test for the OGTT and one-way ANOVA followed by the student–Newman–Keuls post hoc test for the AUC).

The oral glucose tolerance test (OGTT) further confirmed the establishment of glucose intolerance in both experimental models (Figure 3). Following oral glucose administration, STZ-treated animals displayed significantly more elevated blood glucose concentrations throughout the experimental period than their respective control groups. Moreover, glucose concentrations remained markedly elevated after 120 min, indicating impaired glucose clearance.

These alterations were confirmed by analysis of the corresponding areas under the curve (AUCs). Both prediabetic female rats and diabetic male rats exhibited significantly greater AUC values than their respective controls (Figure 3C,D), demonstrating reduced glucose tolerance in both models.

**Figure 4:**
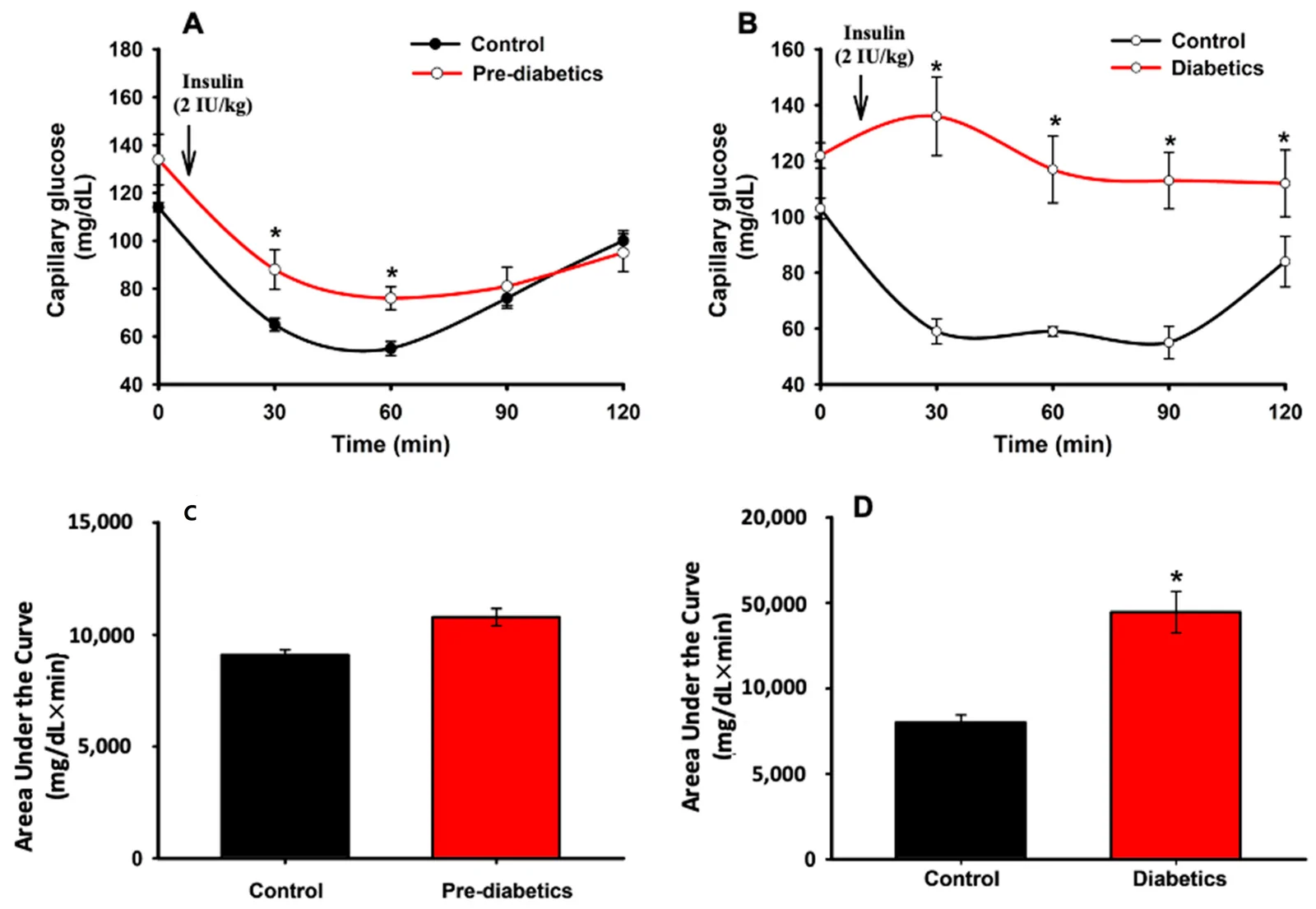
Validation of insulin sensitivity in experimental prediabetes and T2D. Intraperitoneal insulin tolerance tests (IPITTs) performed eight weeks after neonatal streptozotocin administration in (**A**) female PDB rats and (**B**) male rats with T2D. Corresponding areas under the curve (AUCs) are shown in (**C**) female PDB rats and (**D**) male T2D rats. Data are presented as the mean ± SEM *(n* = 6 per group). * = *p* < 0.05 versus the corresponding control group (Two-way repeated-measures ANOVA followed by the student–Newman–Keuls post hoc test for the OGTT and one-way ANOVA followed by the student–Newman–Keuls post hoc test for the AUC).

Insulin sensitivity was subsequently evaluated using the intraperitoneal insulin tolerance test (IPITT). As expected, the metabolic responses differed between female and male animals (Figure 4). Male rats developed a clear insulin-resistant phenotype, characterized by an attenuated hypoglycemic response to exogenous insulin throughout the test period (Figure 4B). Consistent with these findings, the corresponding AUC was significantly higher than that of the control group (Figure 4D).

In contrast, female rats showed only a modest impairment in insulin responsiveness (Figure 4A). Although the glucose disappearance curve suggested a tendency toward reduced insulin responsiveness, the corresponding AUC did not differ significantly from that of control animals (Figure 4C), indicating that insulin resistance remained relatively mild at this stage of disease progression.

Collectively, these findings demonstrate that neonatal STZ administration generated two metabolically distinct experimental models. Female rats developed a phenotype consistent with prediabetes, characterized by moderate fasting hyperglycemia, impaired glucose tolerance, and mild alterations in insulin sensitivity. In contrast, male rats exhibited the characteristic features of experimental T2D, including marked hyperglycemia, severe glucose intolerance, and significant insulin resistance. These well-defined metabolic phenotypes provided an appropriate experimental platform for evaluating the antihyperglycemic activity of freeze-dried *S. stellatus* seeds.

### 2.3. Effects of Freeze-Dried Stenocereus stellatus Seeds on Non-Fasting Hyperglycemia

Following validation of the experimental models, the antihyperglycemic activity of freeze-dried *S. stellatus* seeds was evaluated after oral administration for 14 consecutive days.

A preliminary study performed in male rats with T2D showed that treatment with 100 mg kg^−1^ failed to produce a significant reduction in non-fasting blood glucose concentrations. Therefore, the dose was increased to 200 mg kg^−1^ for subsequent experiments in diabetic animals, whereas the 100 mg kg^−1^ dose was maintained for the prediabetes model.

**Figure 5:**
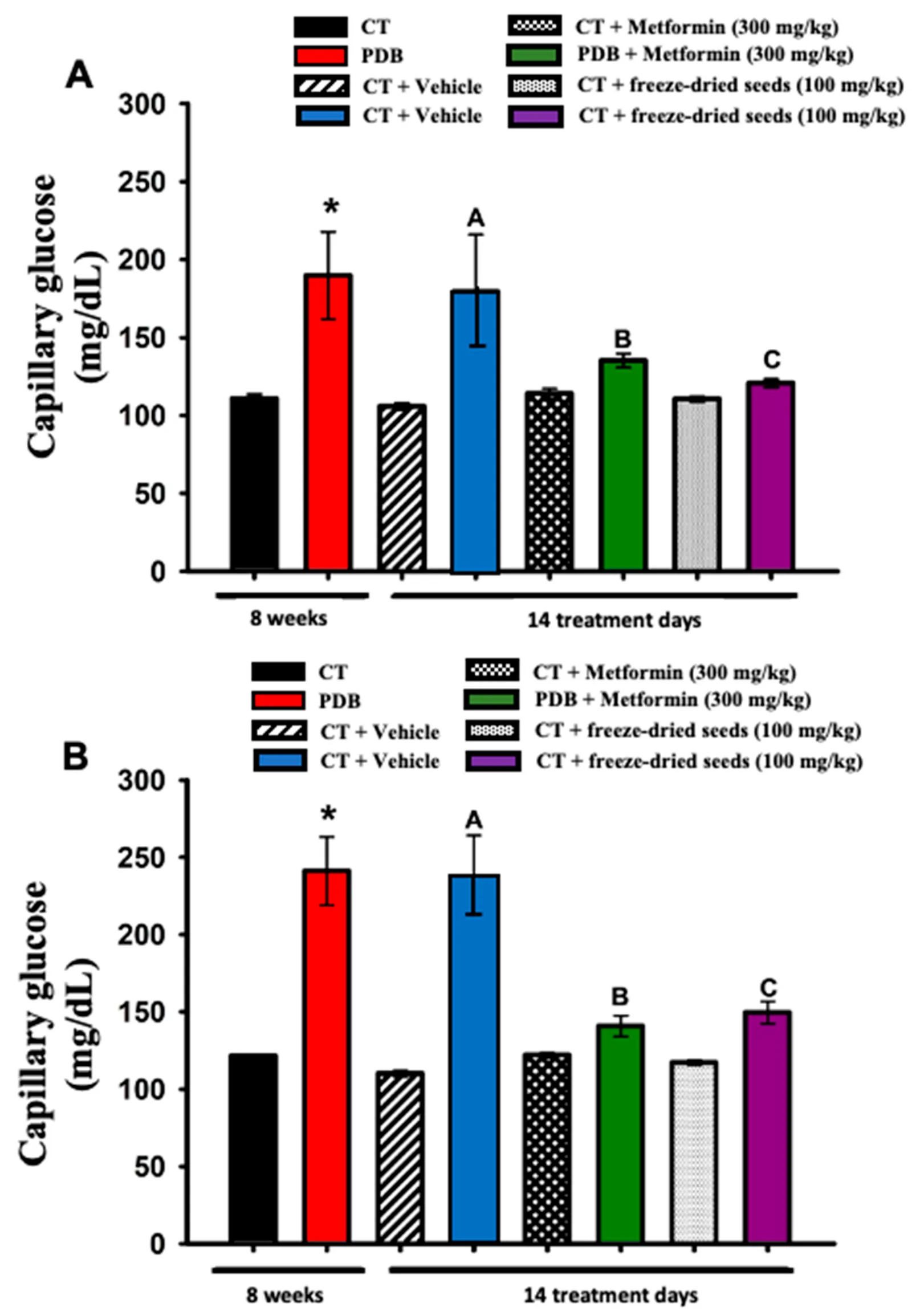
Effects of freeze-dried *S. stellatus* seeds on fasting blood glucose concentrations. Fasting blood glucose after 14 days of oral treatment in (**A**) female rats with PDB receiving freeze-dried seeds (100 mg kg^−1^) and (**B**) male rats with T2D receiving freeze-dried seeds (200 mg kg^−1^). Metformin (300 mg kg^−1^) was included as a positive control. Data are expressed as the mean ± SEM (*n* = 6 per group). Different letters indicate statistically significant differences * = *p* < 0.05 CT 8 weeks vs. PDB or T2D 8 weeks; A = *p* < 0.05, CT + Vehicle vs. PDB or T2D + Vehicle; B = *p* < 0.05, PDB or T2D + Metformin vs. PDB or DB + vehicle; C = *p* < 0.05, PDB or DB + Freeze dried seeds vs. PDB or DB + vehicle; one-way ANOVA followed by the Student–Newman–Keuls post hoc test).

In female rats with prediabetes, administration of freeze-dried *S. stellatus* seeds (100 mg kg^−1^) significantly reduced blood glucose concentrations compared with vehicle-treated prediabetic animals (Figure 5A). A similar reduction was observed in male rats with T2D treated with freeze-dried seeds (200 mg kk^−1^) compared with vehicle-treated diabetic animals (Figure 5B). Metformin was included as a positive control in both models. These results show a glucose-lowering effect of the seed preparation under the experimental conditions evaluated, without implying complete normalization or equivalence between treatments.

A similar antihyperglycemic effect was observed in male rats with T2D. Treatment with freeze-dried seeds (200 mg kg^−1^) significantly decreased fasting blood glucose concentrations compared with diabetic animals receiving vehicle alone (Figure 5B). As in the prediabetic model, the glucose-lowering effect was comparable to that produced by metformin, demonstrating that the freeze-dried *S. stellatus* seeds preparation retained biological activity even in animals with more advanced metabolic impairment.

These findings indicate that 14-day administration of freeze-dried *S. stellatus* seeds attenuated hyperglycemia in both experimental models. Because different doses were used in female PDB and male T2D rats, the magnitude of the responses should not be directly compared between models.

### 2.4. Effects of Freeze-Dried Stenocereus stellatus Seeds on Glucose Tolerance

To determine whether the reduction in no-fasting glycemia was accompanied by improved glucose handling, oral glucose tolerance tests were repeated after completion of the 14-day treatment period.

**Figure 6:**
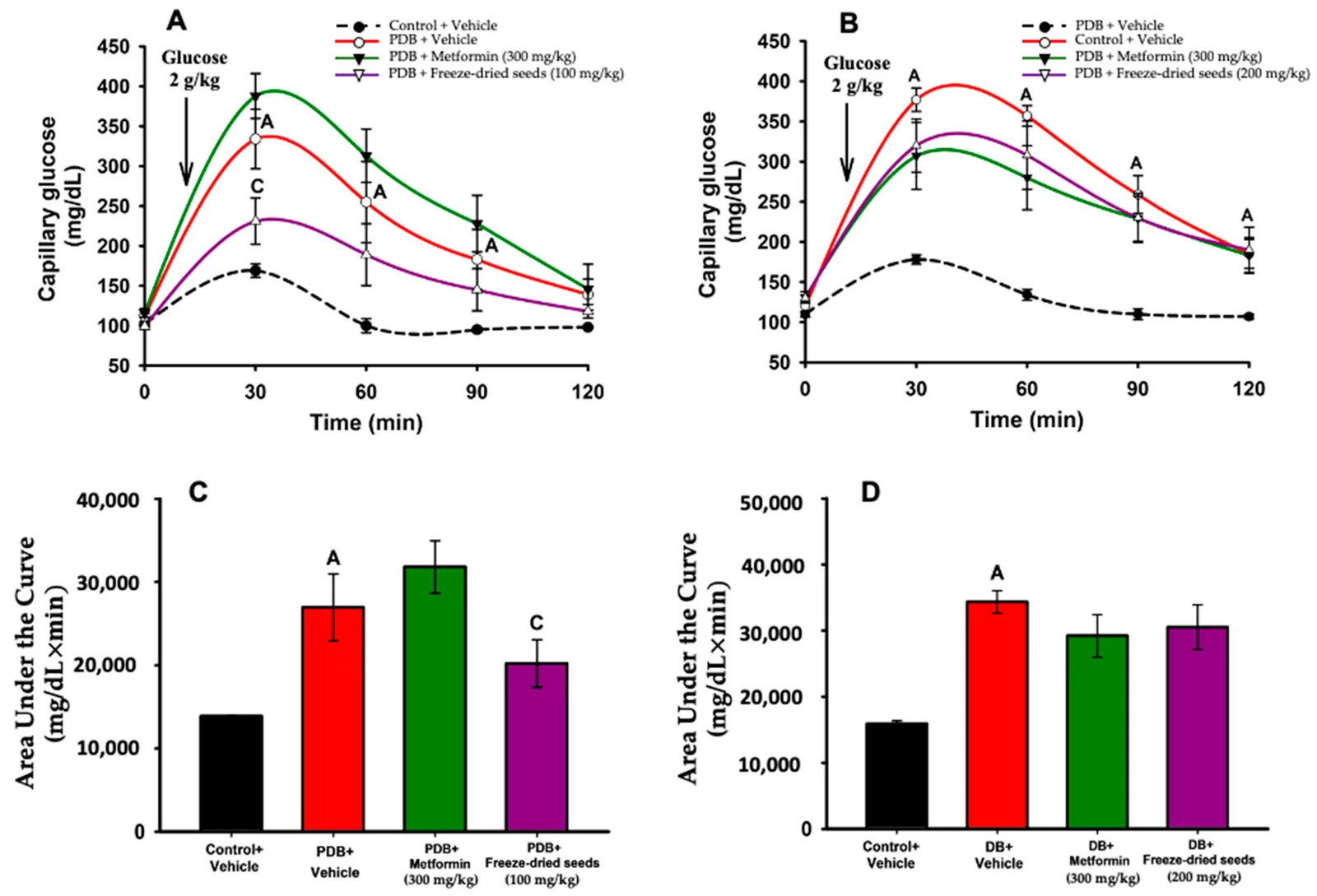
Effects of freeze-dried *S. stellatus* seeds on oral glucose tolerance. Oral glucose tolerance tests (OGTTs) performed after 14 days of treatment in (**A**) female rats with PDB and (**B**) male rats with T2D. Corresponding areas under the curve (AUCs) are presented in (**C**) PDB female rats and (**D**) T2D male rats. Metformin (300 mg kg^−1^) was used as the positive control. A, *p* ˂ 0.05 vs. PDB and T2D vs. Control; C, *p* ˂ 0.05, PDB-freeze-dried *S. stellatus* seeds vs. PDB. Two-way repeated-measures ANOVA followed by the Student–Newman–Keuls post hoc test; data are expressed as the mean ± SEM *(n* = 6 per group).

In female rats with prediabetes, administration of freeze-dried *S. stellatus* seeds markedly improved the glycemic response following oral glucose loading (Figure 6A). Blood glucose concentrations throughout the test were consistently lower than those observed in untreated prediabetic animals, approaching the profile of healthy control rats. Analysis of the corresponding AUC confirmed this improvement, showing a significant reduction in glucose exposure after treatment with freeze-dried seeds compared with the vehicle-treated prediabetic group (Figure 6C). The magnitude of this effect was comparable to that produced by metformin.

In contrast, the response observed in rats with established T2D differed substantially. Although treatment reduced fasting blood glucose concentrations, the overall glucose excursion during the OGTT remained largely unchanged (Figure 6B). Consistent with these observations, no significant differences were detected in the corresponding AUC between freeze-dried seed-treated and vehicle-treated diabetic animals (Figure 6D).

Freeze-dried *S. stellatus* seeds significantly reduced the OGTT glucose excursion in the prediabetes model, whereas no significant improvement in overall OGTT AUC was observed in T2D rats. Because the two models differed in sex and treatment dose, these findings are reported independently and do not establish disease-stage-dependent efficacy.

### 2.5. Effects of Freeze-Dried Stenocereus stellatus Seeds on Fasting Hyperglycemia

To determine whether the antihyperglycemic activity of freeze-dried *S. stellatus* seeds was associated with changes in insulin responsiveness, intraperitoneal insulin tolerance tests (IPITTs) were performed after completion of the 14-day treatment period.

**Figure 7:**
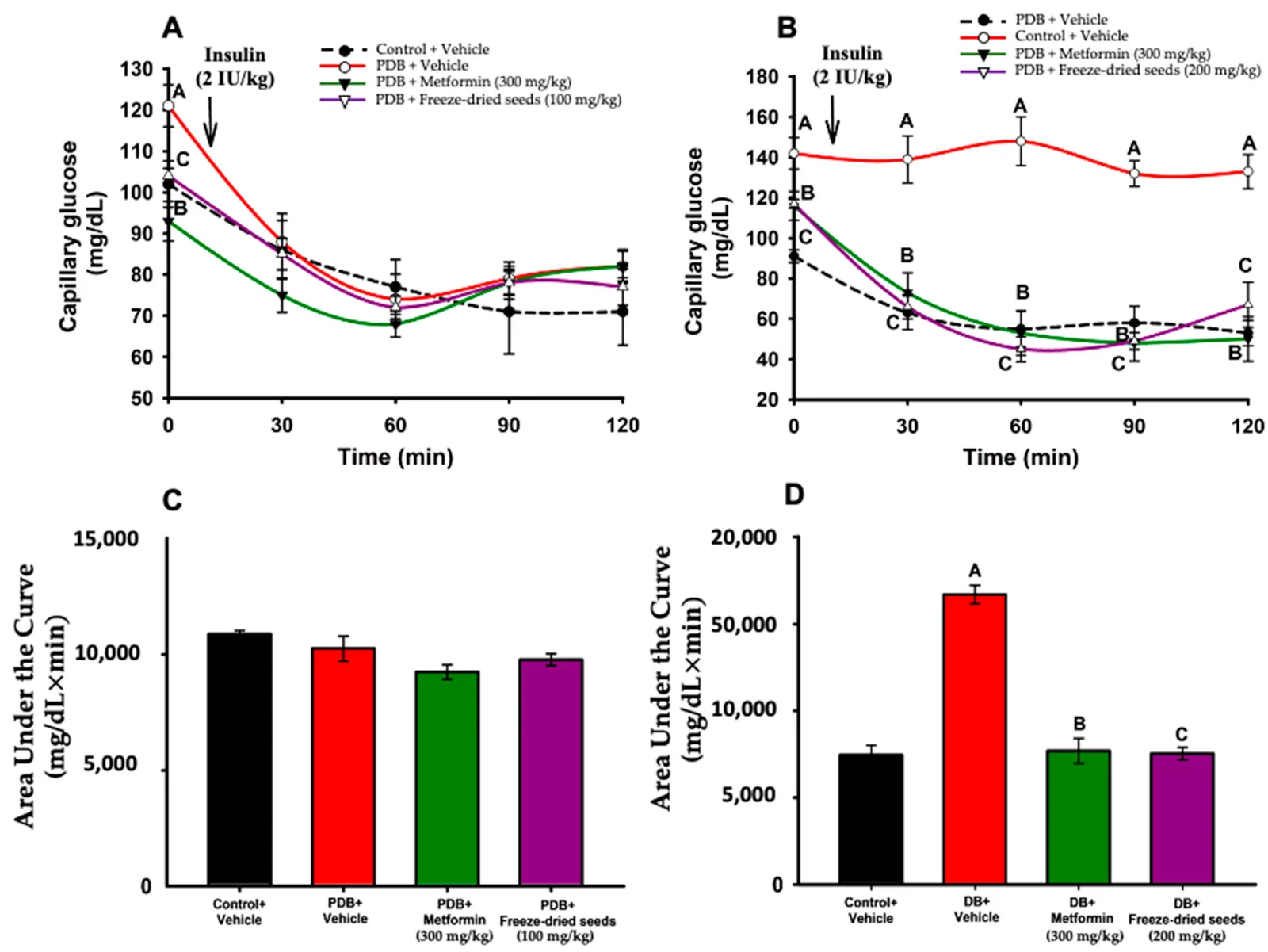
Effects of freeze-dried *S. stellatus* seeds on insulin sensitivity. Intraperitoneal insulin tolerance tests (IPITTs) performed after 14 days of treatment in (**A**) female rats with PDB and (**B**) male rats with T2D. Corresponding areas under the curve (AUCs) are shown in (**C**) PDB female rats and (**D**) T2D rats. Metformin (300 mg kg^−1^) was included as the positive control. A, *p* ˂ 0.05, T2D vs. Control; B, *p* ˂ 0.05, T2D—Metformina vs. T2D; C, *p* ˂ 0.05, T2D—freeze-dried *S. stellatus* seeds vs. T2D. Two-way repeated-measures ANOVA followed by Duncan’s post hoc test. Data are presented as the mean ± SEM (*n* = 6 per group). Different letters indicate statistically significant differences.

In female rats with prediabetes, treatment with freeze-dried seeds (100 mg kg^−1^) did not significantly modify the glucose profile or AUC during the IPITT compared with vehicle-treated prediabetic animals (Figure 7A,C). In male rats with T2D, freeze-dried seeds (200 mg kg^−1^) enhanced the glucose-lowering response to exogenous insulin and reduced the corresponding AUC compared with vehicle-treated diabetic animals (Figure 7B,D). Because fasting insulin concentrations were not measured, these findings are described as changes in insulin responsiveness during the IPITT rather than as direct measurements of whole-body insulin responsiveness.

In contrast, a markedly different response was observed in male rats with established T2D. Vehicle-treated diabetic animals exhibited a blunted hypoglycemic response following insulin administration, confirming the presence of insulin resistance (Figure 7B). Treatment with freeze-dried *S. stellatus* seeds (200 mg kg^−1^) significantly enhanced the glucose-lowering response to exogenous insulin, producing a profile comparable to that observed in metformin-treated animals.

Analysis of the corresponding AUC supported these observations. Diabetic rats receiving freeze-dried seeds exhibited a significant reduction in AUC compared with vehicle-treated diabetic animals (Figure 7D), indicating improved insulin responsiveness. The magnitude of this improvement was similar to that produced by metformin.

Collectively, these results demonstrate that freeze-dried *S. stellatus* seeds exert distinct metabolic effects depending on the stage of disease progression. In prediabetes, treatment primarily restored fasting glycemia and glucose tolerance, whereas in established T2D the principal therapeutic effect was an improvement in insulin sensitivity accompanied by a significant reduction in fasting hyperglycemia.

Taken together, the biological evaluation demonstrated that freeze-dried *S. stellatus* seeds exerted significant antihyperglycemic activity in both experimental models. In prediabetic rats, treatment normalized fasting blood glucose concentrations and restored glucose tolerance without significantly modifying insulin sensitivity. Conversely, in rats with established T2D, the freeze-dried seed preparation significantly reduced fasting hyperglycemia and improved insulin responsiveness, whereas glucose tolerance remained largely unaffected. These findings suggest that the metabolic actions of freeze-dried *S. stellatus* seeds are stage-dependent and may involve complementary mechanisms that differentially influence glucose homeostasis during the progression from prediabetes to overt diabetes.

## 3. Discussion

### 3.1. Principal Findings and Biological Significance

The present study demonstrates that freeze-dried *S. stellatus* seeds possess significant antihyperglycemic activity in experimental models representing two distinct stages of glucose dysregulation, namely prediabetes and T2D. Comprehensive phytochemical characterization revealed that the freeze-dried seeds contain a chemically diverse profile of flavonoids, betalains, phenolic compounds, hydrolysable tannins, and other secondary metabolites, while in vivo administration significantly improved glucose homeostasis through stage-dependent metabolic responses. Specifically, treatment restored fasting hyperglycemia and glucose tolerance in prediabetic animals, whereas in rats with established T2D it reduced fasting hyperglycemia and significantly improved insulin sensitivity. Together, these findings indicate that the biological activity of freeze-dried *S. stellatus* seeds extends beyond simple glucose lowering and instead involves coordinated regulation of complementary metabolic processes associated with the progression of diabetes.

Natural products are increasingly recognized as valuable therapeutic resources because they contain structurally diverse phytochemicals capable of simultaneously modulating multiple molecular pathways involved in chronic diseases. This concept, commonly referred to as network pharmacology or multitarget pharmacology, has become one of the principal paradigms underlying the development of botanical therapeutics for complex disorders such as T2D, where oxidative stress, chronic inflammation, mitochondrial dysfunction, endothelial impairment, and insulin resistance develop simultaneously and reinforce one another [14,15,16,17]. Rather than representing a limitation, the chemical complexity identified in freeze-dried *S. stellatus* seeds may therefore constitute one of its principal biological advantages.

An additional strength of the present study is that it combines detailed phytochemical characterization with functional evaluation in two experimental models representing different stages of disease progression. Most previous investigations involving *Stenocereus* spp. have focused primarily on fruit composition, antioxidant activity, betalain content, nutritional value, or ethnobotanical relevance, whereas comparatively little attention has been devoted to the seeds despite representing approximately one-third of the edible fruit biomass [18,19,20,21]. Furthermore, earlier biological studies have largely evaluated crude fruit preparations or individual antioxidant properties without establishing a direct relationship between phytochemical composition and metabolic efficacy during the progression from prediabetes to overt diabetes. By integrating chromatographic profiling with physiological evaluation in vivo, the present work substantially expands current knowledge regarding the biological potential of *S. stellatus* seeds.

An important conceptual aspect arising from this research is that, in addition to being a source of bioactive molecules, the phytochemical matrix of freeze-dried *S. stellatus* seeds and the synergy of multiple compounds may contribute to the observed therapeutic effect; however, further studies are required to elucidate this. Freeze-drying is particularly relevant in this context because it minimizes thermal degradation and oxidative decomposition of thermolabile compounds, thereby preserving the native phytochemical architecture of the seeds. Maintaining this natural chemical diversity may be essential for conserving biological efficacy, since increasing evidence indicates that secondary metabolites frequently influence the stability, bioavailability, absorption, and biological activity of major phytochemicals through synergistic interactions that cannot be reproduced by isolated compounds [18,22,23,24].

Collectively, these observations provide a conceptual framework for interpreting the biological responses observed throughout the present investigation. Rather than supporting a classical single-target pharmacological mechanism, the results suggest that freeze-dried *S. stellatus* seeds function as a multitarget phytochemical system capable of modulating several interconnected pathways involved in glucose homeostasis. This systems-oriented perspective is increasingly recognized in nutritional pharmacology and provides a biologically coherent explanation for the stage-dependent metabolic responses observed in both prediabetic and diabetic animals.

### 3.2. Phytochemical Composition Supports the Antihyperglycemic Activity of Freeze-Dried Stenocereus stellatus Seeds

Although the fruits of *Stenocereus* spp. have been extensively investigated because of their high nutritional value and antioxidant capacity, the seeds remain a largely underexplored botanical resource despite representing approximately 30–31% of the edible fruit biomass [18,19,20,21]. Previous studies have primarily focused on the pulp, where phenolic compounds, betalains, vitamin C, and other antioxidants have been associated with strong free radical-scavenging activity and potential health benefits [19,20,21]. In contrast, comparatively little information has been available regarding the chemical composition and biological properties of the seeds. The present findings therefore substantially expand current knowledge by demonstrating that freeze-dried *S. stellatus* seeds contain a chemically diverse mixture of flavonoids, betalains, hydrolysable tannins, phenolic compounds, and other secondary metabolites with recognized biological relevance.

Among the identified compounds, quercetin represents one of the most extensively studied dietary flavonoids with demonstrated antidiabetic properties. Numerous experimental studies have shown that quercetin improves glucose homeostasis through activation of AMP-activated protein kinase (AMPK), enhancement of phosphatidylinositol-3 kinase/protein kinase B (PI3K/Akt) signaling, stimulation of GLUT4 translocation, suppression of hepatic gluconeogenesis, and preservation of pancreatic β-cell viability under conditions of oxidative stress [22,23,24,25,26]. Quercetin also attenuates activation of NF-κB and other pro-inflammatory pathways, thereby reducing the production of cytokines such as TNF-α and IL-6 that contribute directly to insulin resistance [23,27]. These complementary biological activities provide a plausible mechanistic basis for the antihyperglycemic effects observed following administration of the freeze-dried seed preparation.

The identification of betanin further strengthens the biological relevance of the phytochemical profile. Betalains constitute characteristic pigments of several cactus fruits and possess potent antioxidant activity in addition to their coloring properties [19,21,25]. Previous investigations have demonstrated that betanin improves glucose metabolism in experimental diabetes by activating AMPK/SIRT1 signaling, suppressing NF-κB-mediated inflammation, preserving pancreatic β-cell integrity, increasing hepatic glycogen storage, and enhancing endogenous antioxidant defenses through activation of Nrf2-dependent pathways [22,23,24,27]. These mechanisms are particularly relevant because oxidative stress is recognized as one of the earliest pathogenic events linking chronic hyperglycemia with progressive β-cell dysfunction and insulin resistance. Consequently, the presence of betanin within the freeze-dried seed matrix may contribute not only to direct antioxidant protection but also to long-term preservation of cellular homeostasis.

In addition to quercetin and betanin, UPLC-QTOF-MS identified other flavonoids and phenolic metabolites that have individually demonstrated beneficial effects on metabolic regulation. Artemetin has been reported to exhibit antioxidant, anti-inflammatory, antihypertensive, endothelial-protective, and α-amylase inhibitory activities, while eriocitrin improves lipid metabolism, attenuates insulin resistance, and enhances β-oxidation through activation of PPARα-dependent pathways [1,2,3,4,28,29,30,31,32,33]. Particularly noteworthy is the recent clinical evaluation of the eriocitrin-based nutraceutical Eriomin^®^, which significantly reduced fasting blood glucose, improved insulin resistance, increased circulating glucagon-like peptide-1 (GLP-1), and decreased systemic inflammation in individuals with prediabetes [33]. Although retusin, resorcinol, eugeniin, and esmeraldic acid have been less extensively investigated in the context of diabetes, available evidence indicates that these compounds possess antioxidant, anti-inflammatory, enzyme-modulating, or cytoprotective properties that could complement the biological activities of the major flavonoids identified in the present study [1,2,3,28,32,33,34,35,36].

Importantly, the therapeutic potential of the freeze-dried seed preparation should not be interpreted as the sum of independent actions exerted by individual metabolites. Increasing evidence indicates that botanical preparations frequently derive their efficacy from phytochemical cooperation, whereby structurally distinct compounds simultaneously regulate complementary molecular targets involved in glucose metabolism, oxidative stress, mitochondrial function, inflammation, endothelial biology, and insulin signaling. Such cooperative interactions represent one of the defining characteristics of complex botanical matrices and distinguish them from conventional single-target pharmacological agents. Consequently, preservation of the native phytochemical composition through freeze-drying may represent an important determinant of biological efficacy, since removal or purification of individual constituents could potentially diminish these cooperative interactions. Within this context, the chemically diverse metabolite profile identified in *S. stellatus* seeds provides a biologically plausible explanation for the antihyperglycemic activity observed in vivo and supports their future development as a standardized nutraceutical or functional food ingredient for metabolic disorders [14,15,16,17,22,23,24].

### 3.3. Metabolic Effects of Freeze-Dried Stenocereus stellatus Seeds in Prediabetes

In the prediabetes model, freeze-dried *S. stellatus* seeds reduced blood glucose concentrations and significantly improved the glycemic response during the OGTT. These findings extend our previous observations in this experimental model [5] and indicate that the seed preparation can influence glucose handling after short-term oral administration. However, the IPITT did not show a significant change in AUC, suggesting that the improvement in glucose tolerance cannot be attributed solely to a measurable change in the response to exogenous insulin under the conditions tested.

The phytochemical profile provides possible candidates for future mechanistic studies, but the present experiments were not designed to establish causal relationships between individual metabolites and the metabolic response. Accordingly, pathways previously associated with flavonoids, phenolic compounds, or betalains should be considered background context rather than mechanisms demonstrated here [22,23,24,25,26,27].

Because the prediabetes and T2D experiments used animals of different sexes and different seed doses, the present data do not support a direct comparison of efficacy between disease stages. The biological findings should therefore be interpreted within each model.

### 3.4. Metabolic Effects of Freeze-Dried Stenocereus stellatus Seeds in Type 2 Diabetes

In the T2D model, treatment with freeze-dried *S. stellatus* seeds reduced hyperglycemia and enhanced the glucose-lowering response to exogenous insulin. In contrast, the overall glucose excursion during the OGTT remained significantly impaired. This pattern indicates a partial metabolic effect rather than restoration of glucose homeostasis.

The reduced IPITT AUC is consistent with improved responsiveness to administered insulin during the test. Nevertheless, fasting insulin was not measured and insulin-based indices such as HOMA-IR or QUICKI could not be calculated. Therefore, the present study does not establish a direct quantitative change in whole-body insulin responsiveness.

Several metabolites tentatively annotated in the seed preparation have been associated in previous studies with pathways involved in glucose metabolism and oxidative stress [1,2,3,4,22,23,24,25,26,27,28,29,30,31,32,33]. These reports provide biological context but do not demonstrate that the same mechanisms operated in the present experiments [34,35,36]. Direct mechanistic studies will be required to determine which constituents and pathways contribute to the observed effects.

Taken together, the T2D findings show that the freeze-dried seed preparation reduced hyperglycemia and altered the response to exogenous insulin without significantly improving overall oral glucose tolerance [14,25,37,38]. This distinction is important when defining the scope of the antihyperglycemic activity observed.

### 3.5. Translational Relevance and Future Perspectives

*S. stellatus* seeds are an underused component of an edible cactus fruit and may represent a source of phytochemicals for further investigation [39,40,41,42,43,44]. Freeze-drying is useful for preserving plant material while limiting thermal degradation, but the present study did not evaluate product stability, bioavailability, long-term safety, or clinical efficacy. Therefore, the current findings should be considered preclinical evidence of antihyperglycemic activity rather than evidence supporting a nutraceutical indication.

Future studies should quantify the major metabolites using authentic standards and validated analytical methods, confirm structural assignments, evaluate relevant molecular endpoints, and assess longer treatment periods and safety. These steps will be necessary before the potential use of freeze-dried *S. stellatus* seeds as a functional food ingredient or nutraceutical can be established.

## 4. Materials and Methods

### 4.1. Plant Material

Ripe fruits of *S. stellatus* were harvested in September 2017 from orchards located near San Juan Joluxtla, Cosoltepec, Oaxaca, Mexico. Fruits were selected according to external maturity indicators, including bright peel coloration, easy spine detachment, and the absence of visible mechanical damage or disease symptoms. After harvesting, the fruits were transported to the laboratory in Mexico City and stored at 11 °C until processing. Fruits were washed with a disinfectant solution containing 200 ppm active chlorine, after which the seeds were manually separated from the pulp and freeze-dried using a FreeZone 2.5 lyophilizer (Labconco, Kansas City, MO, USA).

### 4.2. Preparation of the Freeze-Dried Stenocereus stellatus Seed

An aqueous solution was prepared by suspending 1 g of freeze-dried seed powder in 10 mL of deionized water (Simplicity Water Purification System, Merck Millipore, Darmstadt, Germany). The suspension was placed in a 50 mL amber glass flask and stirred at 100 rpm for 1 h using an orbital shaker (Digital Orbital Shaker, ICB, Zapopan, Jalisco, Mexico). The mixture was subsequently incubated at room temperature for 72 h under light-protected conditions to facilitate extraction of water-soluble phytochemicals. The extract was filtered through a 0.45 µm nitrocellulose membrane (Merck Millipore, Darmstadt, Germany) and stored at 4–8 °C until analysis.

### 4.3. Determination of the Total Phenolics Content

Determination of the total phenolics content in the aqueous extract was evaluated using the Folin–Ciocalteu reaction, which is based on the reduction of phosphomolybdic-phosphotungstic acid complexes under alkaline conditions, producing a characteristic blue chromophore [42,45].

Briefly, 25 µL of the aqueous extract was mixed with 100 µL of Folin–Ciocalteu reagent (2 N; Sigma-Aldrich, St. Louis, MO, USA) in a 96-well microplate and shaken at 400 rpm for 5 min (MS3 Digital Microplate Shaker, IKA Works Inc., Wilmington, NC, USA). Subsequently, 75 µL of 20% (*w*/*v*) anhydrous sodium carbonate (Sigma-Aldrich) was added, and the plate was shaken again for 5 min. After incubation in the dark for 2 h at room temperature, the development of a blue coloration was considered indicative of the presence of phenolic compounds [42,45].

### 4.4. UPLC Analysis of Phenolic Compounds

Phenolic compounds present in the aqueous extract (10%, *w*/*v*) were analyzed using an ACQUITY UPLC H-Class Plus Bio System (Waters Corporation, Milford, CT, USA) equipped with a photodiode array detector. Chromatographic separation was performed on an ACQUITY UPLC CSH C18 column (100 × 2.1 mm, 1.7 µm, 130 Å; Waters).

Samples (10 µL) were maintained at 10 °C prior to injection. The mobile phase consisted of acetonitrile (HPLC grade; Sigma-Aldrich, Urbana, IL, USA) and 85% phosphoric acid (JT Baker, Xalostoc, Estado de México, Mexico) diluted in HPLC-grade water (35:65, *v*/*v*) and adjusted to pH 2.0. Separation was carried out at a flow rate of 0.30 mL min^−1^ over 12 min, and chromatograms were recorded at 210 nm. The chromatographic method was adapted from Santos et al. [43].

### 4.5. Fourier Transform Infrared (FTIR) Spectroscopy

Chromatographic fractions displaying well-defined peaks after UPLC separation were individually subjected to the Folin–Ciocalteu reaction to confirm the presence of phenolic compounds. Fractions showing positive reactions were pooled according to their chromatographic profiles, concentrated under a gentle stream of nitrogen at room temperature, and analyzed by Fourier transform infrared spectroscopy (FTIR).

Infrared spectra were acquired using a Nicolet 4700 FTIR spectrometer (Thermo Fisher Scientific, Waltham, MA, USA). Spectral analysis was performed to identify characteristic functional groups associated with phenolic compounds, following the methodology described by Santos et al. [43].

### 4.6. Identification of Betanin

Fractions C, D, and E obtained after UPLC separation were analyzed for the presence of betanin, the principal betalain pigment responsible for the characteristic red coloration of several cactus fruits.

Briefly, 25 µL of each fraction was mixed with 500 µL of 0.1 M acetic acid prepared with deionized water and incubated for 15 min at room temperature under light-protected conditions. Subsequently, 500 µL of 0.1 M antimony (III) chloride dissolved in chloroform was added, and the reaction mixture was incubated at 100 °C for 15 min using a dry heating block (DS200, LabGenius, London, UK). The appearance of a characteristic red coloration that changed to blue under long-wave ultraviolet light (365 nm) was considered indicative of the presence of betanin, according to the procedure described by Mohammed et al. [44].

### 4.7. UPLC-QTOF Analysis of the Freeze-Dried Seed Preparation

Because the biological studies were conducted using oral doses of 100 and 200 mg kg^−1^ of freeze-dried seed suspended in water (1 mL per 100 g body weight), the phytochemical characterization by UPLC-QTOF-MS was performed using a 1% (*w*/*v*) aqueous preparation to reproduce the formulation administered to the experimental animals.

Freeze-dried seed powder was suspended in deionized water (10 mg mL^−1^), protected from light, and stored at 4 °C for 72 h before analysis.

Chromatographic separation was performed using an ACQUITY UPLC system (Waters Corporation, Milford, MA, USA) equipped with a BEH C18 reverse-phase column (100 × 2.1 mm, 1.7 µm). Mobile phase A consisted of LC-MS-grade water containing 0.01% formic acid, whereas mobile phase B consisted of LC-MS-grade acetonitrile containing 0.01% formic acid. The flow rate was 0.30 mL min^−1^ and the injection volume was 10 µL.

The gradient program was as follows: 96% A (0–9 min), 80% A (10–14 min), 65% A (15–19 min), 100% B (20–29 min), followed by re-equilibration to the initial conditions. Chromatographic conditions were based on those previously described by Oidor-Chan et al. [5].

### 4.8. UPLC-QTOF-MSE Acquisition and Metabolite Annotation

Mass spectrometric analysis was performed using an Xevo G2-XS quadrupole time-of-flight mass spectrometer (Waters Corporation, Milford, MA, USA) equipped with an electrospray ionization (ESI) source operating in negative-ion mode. Data acquisition and instrument control were performed using UNIFI software, version 1.9 (Waters Corporation, Milford, MA, USA). The capillary voltage was 2.0 kV; nitrogen was used as desolvation and cone gas at 400 and 50 L h^−1^, respectively; source and desolvation temperatures were 150 and 800 °C, respectively.

Leucine-enkephalin (200 pg mL^−1^ in acetonitrile/water, 50:50, containing 0.1% formic acid) was used as lock-mass reference. External calibration was performed with 0.5 mM sodium formate in 2-propanol/water (90:10) over *m*/*z* 50–1200. Data were acquired in MSE mode using 6 eV for the low-energy function and a 20–30 eV collision-energy ramp for the elevated-energy function, with a cone voltage of 20 V.

Raw data were processed in Progenesis QI (Nonlinear Dynamics, Newcastle upon Tyne, UK) for feature extraction and alignment. Candidate annotations were evaluated using accurate precursor mass, proposed elemental composition, isotopic pattern, and ions associated with the elevated-energy MSE trace. Database/software matches were treated as candidate assignments rather than structural confirmation. A precursor and fragment mass tolerance of 5 ppm was used in the Pro-genesis workflow. Because authentic standards were not analyzed, annotations were reported as putative or formula/isomer-level assignments according to the strength of the available evidence. Relative peak-area values were used only as signal-abundance descriptors and not as absolute metabolite concentrations.

Data were acquired in MSE mode using a low collision energy of 6 eV and a high collision energy ramp of 20–30 eV with a cone voltage of 20 V. All solvents were LC-MS grade. Raw data were processed using software Progenesis QI, version 3.1.9211.37750 (Nonlinear Dynamics, Newcastle upon Tyne, UK) for feature extraction, alignment, and tentative metabolite identification [46].

### 4.9. Experimental Animals and Induction of Prediabetes and Type 2 Diabetes

All animal procedures were conducted in accordance with the Mexican Official Standard for the Care and Use of Laboratory Animals (NOM-062-ZOO-1999, SAGARPA, Mexico) and the Guide for the Care and Use of Laboratory Animals [47]. The experimental protocol was reviewed and approved by the Institutional Ethics Committee and complied with national regulations governing animal experimentation.

Neonatal Wistar rats (2–3 days old) of both sexes were used to establish experimental models of prediabetes (female rats) and type 2 diabetes (male rats). Prediabetes (PDB) and T2D were induced by a single intraperitoneal injection of streptozotocin (STZ, 70 mg kg^−1^) dissolved in freshly prepared 0.1 M citrate buffer (pH 4.5). Age-matched control animals received an equivalent volume of citrate buffer (1 mL per 100 g body weight).

**Figure 8:**
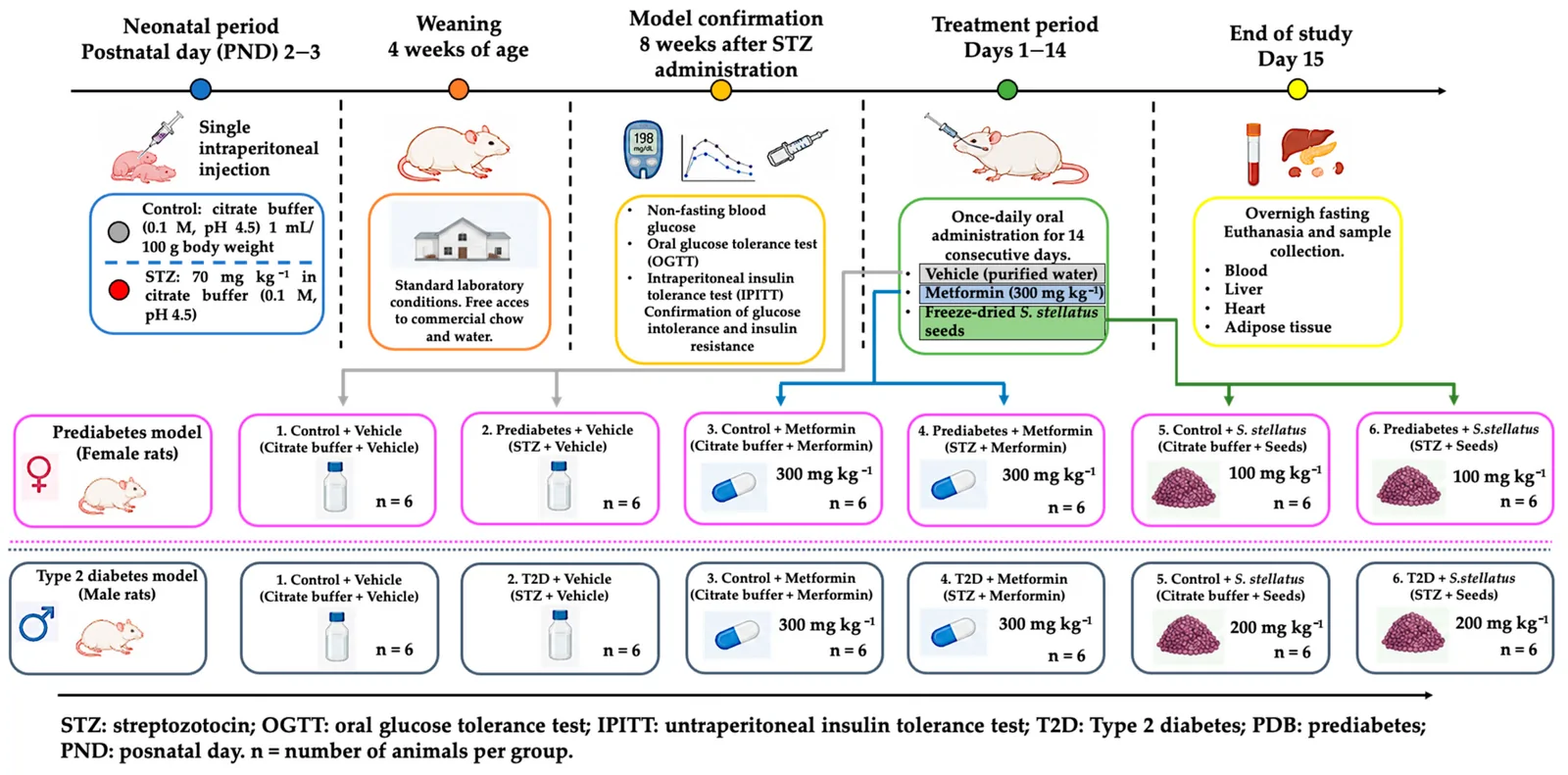
Experimental timeline and treatment protocol. Animals were randomly assigned to the corresponding experimental groups one day before treatment initiation. Baseline body weight and fasting blood glucose were recorded on Day 0, followed by once-daily oral administration of vehicle (purified water), metformin, or freeze-dried *S. stellatus* seeds for 14 consecutive days. Female rats in the prediabetes model received freeze-dried *S. stellatus* seeds at 100 mg kg^−1^, whereas male rats in the type 2 diabetes model received 200 mg kg^−1^. Metformin was administered at 300 mg kg^−1^ in both experimental models. Following the final treatment, animals were fasted overnight, and biological samples were collected on Day 15 for biochemical and complementary analyses.

Animals were weaned at four weeks of age and maintained under standard laboratory conditions with free access to commercial chow and water. Eight weeks after STZ administration, non-fasting blood glucose concentrations, oral glucose tolerance tests (OGTT), and intraperitoneal insulin tolerance tests (IPITT) were performed to confirm the establishment of glucose intolerance and insulin resistance before initiating pharmacological treatments [48] (Figure 8).

### 4.10. Oral Glucose Tolerance Test (OGTT)

Oral glucose tolerance was evaluated eight weeks after STZ administration and repeated at the end of the treatment period.

Animals were fasted for 14 h with free access to water. Baseline blood glucose concentrations (time 0) were measured from the tail vein using an Accu-Chek Active glucometer (Roche Diabetes Care, Indianapolis, IN, USA), after which body weight was recorded.

A glucose solution (2 g kg^−1^) prepared in purified water was administered orally using a stainless-steel gastric gavage needle at a volume of 1 mL per 100 g body weight.

Capillary blood glucose concentrations were subsequently determined at 30, 60, 90, and 120 min after glucose administration using approximately 1 µL of blood obtained from the tail vein.

Results are expressed as the mean ± standard error of the mean (SEM). Glucose tolerance was quantified by calculating the area under the glucose concentration-time curve (AUC) using the trapezoidal method [5].

### 4.11. Intraperitoneal Insulin Tolerance Test (IPITT)

Insulin sensitivity was assessed eight weeks after STZ administration and again after completion of the treatment protocol (Figure 8).

Animals were fasted for 6 h before the experiment. Baseline blood glucose concentrations were determined from the tail vein immediately before insulin administration.

Neutral protamine Hagedorn insulin (Insulex^®^, PiSA Biotec, Guadalajara, Jalisco, Mexico) was administered intraperitoneally at a dose of 2 IU kg^−1^.

Blood glucose concentrations were measured at 30, 60, 90, and 120 min following insulin injection using the same glucometer and sampling procedure described for the OGTT.

Data are presented as the mean ± SEM, and insulin sensitivity was evaluated by calculating the area under the glucose concentration-time curve (AUC) using the trapezoidal method.

### 4.12. Evaluation of the Antihyperglycemic Activity of Freeze-Dried Stenocereus stellatus Seeds

The antihyperglycemic activity of freeze-dried *S. stellatus* seeds was evaluated after confirmation of the experimental models of prediabetes and T2D.

Treatments were administered orally once daily for 14 consecutive days. Experimental groups consisted of six animals each (*n* = 6).

Metformin was included as the positive control. The doses of metformin and freeze-dried seed preparations were selected according to previous experimental studies [5,13].

At the end of the 14-day treatment period, all animals were subjected to a second OGTT and IPITT following the same procedures described above. Non-fasting blood glucose concentrations and body weight gain were also determined to evaluate the effects of treatment on glycemic control and metabolic status (Figure 8).

The primary endpoints included changes in blood glucose concentrations, glucose tolerance, glucose-lowering response to exogenous insulin during the IPITT, and body weight. The antihyperglycemic activity of the freeze-dried seed preparation was assessed by comparison with the corresponding vehicle-treated disease group and with metformin as a positive control.

### 4.13. Statistical Analysis

Statistical analyses were performed using SigmaPlot software version 15.0 (Systat Software Inc., San Jose, CA, USA). Data are presented as the mean ± standard error of the mean (SEM). Statistical significance was established at *p* < 0.05.

Baseline oral glucose tolerance test (OGTT) and intraperitoneal insulin tolerance test (IPITT) curves were analyzed using two-way repeated-measures analysis of variance (ANOVA) followed by the Student–Newman–Keuls multiple-comparison test.

The corresponding areas under the curve (AUCs) obtained from baseline OGTT and IPITT were compared using one-way ANOVA followed by the Student–Newman–Keuls post hoc test.

Following the 14-day treatment period, fasting and non-fasting blood glucose concentrations, body weight gain, and AUC values obtained from the OGTT and IPITT were analyzed using two-way repeated-measures ANOVA followed by the Student–Newman–Keuls multiple-comparison test or Duncan’s method, respectively. Differences were considered statistically significant when *p* < 0.05. Normality of residuals was formally evaluated using the Shapiro–Wilk test. When raw datasets deviated significantly from a normal distribution, appropriate mathematical transformations (specifically, natural logarithm +1) were applied to achieve normality and equality of variances prior to performing parametric ANOVA testing. For graphical representation and biological clarity, descriptive data in the figures and tables are reported as the original (non-transformed) mean ± SEM values.

## 5. Study Limitations

Although the present study provides comprehensive evidence supporting the antihyperglycemic activity of freeze-dried *S. stellatus* seeds, several limitations should be considered when interpreting the findings.

First, metabolite assignments obtained by UPLC-QTOF-MSE are putative. Authentic reference standards were not analyzed, and the strength of the available accurate-mass, isotopic, and elevated-energy evidence differed among chromatographic features. In particular, some features could only be assigned at the molecular-formula or isomer-candidate level. Targeted MS/MS and comparison with authentic standards, and where necessary complementary spectroscopic methods, will be required for definitive structural confirmation.

Second, the UPLC-QTOF-MSE analysis was intended for phytochemical profiling rather than validated quantification. Relative peak areas should not be interpreted as absolute concentrations. Quantification of major metabolites will require authentic standards, compound-specific calibration curves, and analytical validation including linearity, precision, recovery, LOD, and LOQ.

Third, the biological experiments included six animals per group and no prospective power calculation was performed. The prediabetes and T2D models also differed in sex and seed dose and were therefore analyzed as separate experiments; the study was not designed to establish disease-stage-dependent efficacy. In addition, fasting insulin was not measured, preventing calculation of HOMA-IR, QUICKI, or related indices.

Fourth, the metabolic characterization was limited to blood glucose, OGTT, IPITT, and body-weight-related endpoints. Lipid profile, hepatic biochemical markers, pancreatic histology, and direct molecular measurements were not included. Consequently, mechanistic interpretations involving insulin-signaling, antioxidant, inflammatory, or mitochondrial pathways cannot be established from the present data.

An additional limitation is that the UPLC-QTOF-MS analysis was primarily designed for phytochemical profiling rather than targeted quantification. Therefore, the peak-area ratios reported for the annotated metabolites represent relative signal abundance and should not be interpreted as absolute concentrations. Quantitative determination of the major metabolites using authentic standards and compound-specific calibration curves will be necessary to establish their concentrations in the freeze-dried seed preparation and to better relate chemical composition to biological activity.

Finally, treatment lasted 14 days, and long-term efficacy, safety, pharmacokinetics, bioavailability, and interactions with the gut microbiota were not evaluated. These issues should be addressed before translational or nutraceutical applications are considered.

Despite these limitations, the present investigation integrates comprehensive phytochemical characterization with functional evaluation in two complementary models representing different stages of glucose dysregulation. The consistency between the chemical profile and the observed biological responses provides a robust experimental foundation for future mechanistic studies and supports continued investigation of freeze-dried *S. stellatus* seeds as a promising source of bioactive compounds for metabolic health.

## 6. Conclusions

The present study expands the phytochemical profile of freeze-dried *S. stellatus* seeds and demonstrates antihyperglycemic activity in experimental models of prediabetes and T2D. UPLC-QTOF-MSE detected several chromatographic features that could be annotated at different confidence levels; because authentic standards were not used, these assignments remain putative and require further confirmation.

In prediabetic rats, the seed preparation reduced blood glucose and improved oral glucose tolerance. In T2D rats, it reduced hyperglycemia and enhanced the glucose-lowering response to exogenous insulin, while overall glucose tolerance remained impaired. Because the models differed in sex and dose, these responses should not be interpreted as evidence of disease-stage-dependent efficacy.

The present findings support further investigation of *S. stellatus* seeds as a source of bioactive phytochemicals. Quantitative chemical analysis, structural confirmation, mechanistic experiments, longer-term safety studies, and ultimately clinical evaluation will be required before functional-food or nutraceutical applications can be proposed.

## Figures and Tables

**Table 1:** UPLC-QTOF-MSE features and putative metabolites detected in the aqueous preparation of freeze-dried *Stenocereus stellatus* seeds.

No.	Relativepeak Area (%)	RT (min)	Ion	Theorical *m*/*z*	Observed *m*/*z* (Error, ppm)	Associated High-Energy Ions (*m*/*z*)	Formula	Metabolites	Evidence/Confidence	Class
1	100	0.82	[M-H]^−^	387.1085	387.1079 (−1.7)	179.0444; 221.0551; 341.1004	C_20_H_20_O_8_	Artemetin	Putative; exact-mass/isotope support; UNIFI concordant; no authentic standard.	O-methylated flavone
2	56.37	6.85	[M-H]^−^	517.1517	517.1537 (+3.7)	146.9516; 174.9441; 193.0386; 295.0720; 356.8221	C_30_H_22_N_4_O_5_	Esmeraldic acid candidate	Low-confidence putative; unusual plant assignment; no authentic standard.	Candidate phenazine-related annotation
3	68.28	18.49	[M-H]^−^	357.0980	357.0963 (−4.69)	No diagnostic product ions confidently assigned	C_19_H_18_O_7_	Retusin candidate	Low-confidence putative; multiple C_19_H_18_O_7_ isomers; UNIFI mass mismatch.	Flavonoid candidate
4	97.72	26.72	[M-H]^−^	595.1668	595.1658 (−1.79)	No diagnostic product ions confidently assigned	C_27_H_32_O_15_	Eriocitrin candidate	Low-confidence putative; Progenesis fragmentation score 4.07; multiple isomers; UNIFI mass mismatch.	Flavonoid glycoside candidate
5	96.29	26.77	[M-H]^−^	937.0953	937.0909 (−4.7)	Predominantly near-precursor/isotopic ions; no diagnostic fragments	C_41_H_30_O_26_	Eugeniin	Formula/isomer-level annotation; eugeniin and davidiin candidates; no diagnostic fragments.	Hydrolysable tannin candidate
6	100	26.85	[M-H]^−^	109.0295	109.0292 (−3.1)	99.9926; 103.0018; 107.9947 (non-diagnostic)	C_6_H_6_O_2_	Resorcinol	Formula-level annotation; multiple C_6_H_6_O_2_ isomers; resorcinol is one candidate.	Benzenediol isomer candidate

## Data Availability

The authors confirm that the data supporting the findings of this study are available within the article. The datasets of this study are available from the corresponding authors upon reasonable request.

## Supplementary Materials

The following supporting information can be downloaded at: https://www.mdpi.com/article/10.3390/molecules31172964/s1.

## Abbreviations

The following abbreviations are used in this manuscript: Type 2 diabetes (T2D), Insulin tolerance (IT), oral glucose tolerance tests (OGTT), Streptozotocin (STZ).
