# Supplementary material for: Phytochemical Characterization of Freeze-Dried Stenocereus stellatus Seeds and Their Antihyperglycemic Activity in Experimental Prediabetes and Type 2 Diabetes
Source: Molecules. 2026 Aug 25;31(17):2964. doi: 10.3390/molecules31172964 (PMC13567401; doi:10.3390/molecules31172964)

Representative UPLC chromatograms and low- and high-energy MS^E spectra of the aqueous extract prepared from freeze-dried Stenocereus stellatus seeds.

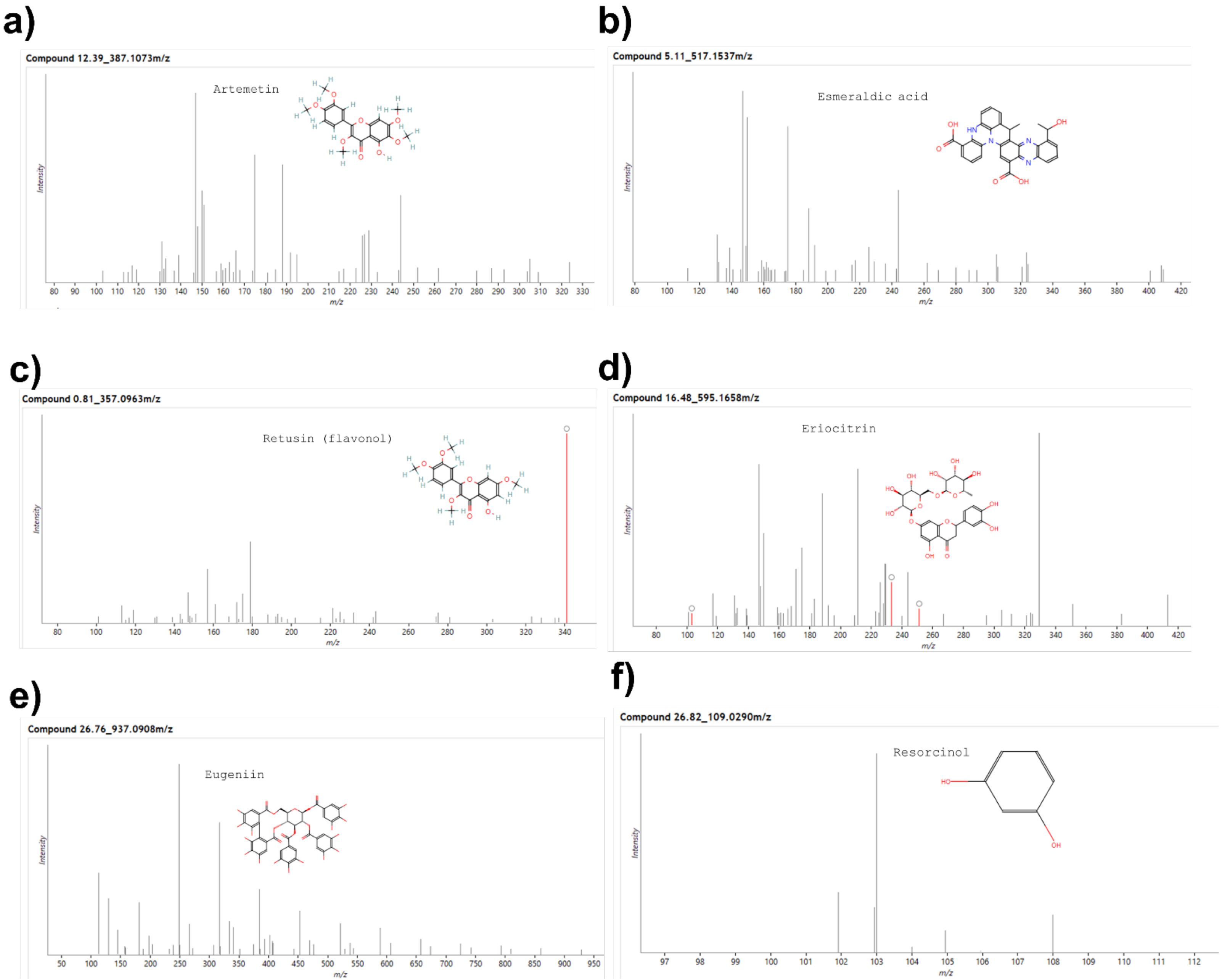

Supplement: Supplementary file 1 [file molecules-31-02964-s001.zip › Supplementary Figure S2 .pdf]
