# Supplementary material for: Phytochemical Characterization of Freeze-Dried Stenocereus stellatus Seeds and Their Antihyperglycemic Activity in Experimental Prediabetes and Type 2 Diabetes
Source: Molecules. 2026 Aug 25;31(17):2964. doi: 10.3390/molecules31172964 (PMC13567401; doi:10.3390/molecules31172964)

Retention times and corresponding low- and high-energy MS^E spectra of phytochemicals identified in the aqueous extract prepared from freeze-dried *Stenocereus stellatus* seeds by UPLC-QTOF-MS.

a)

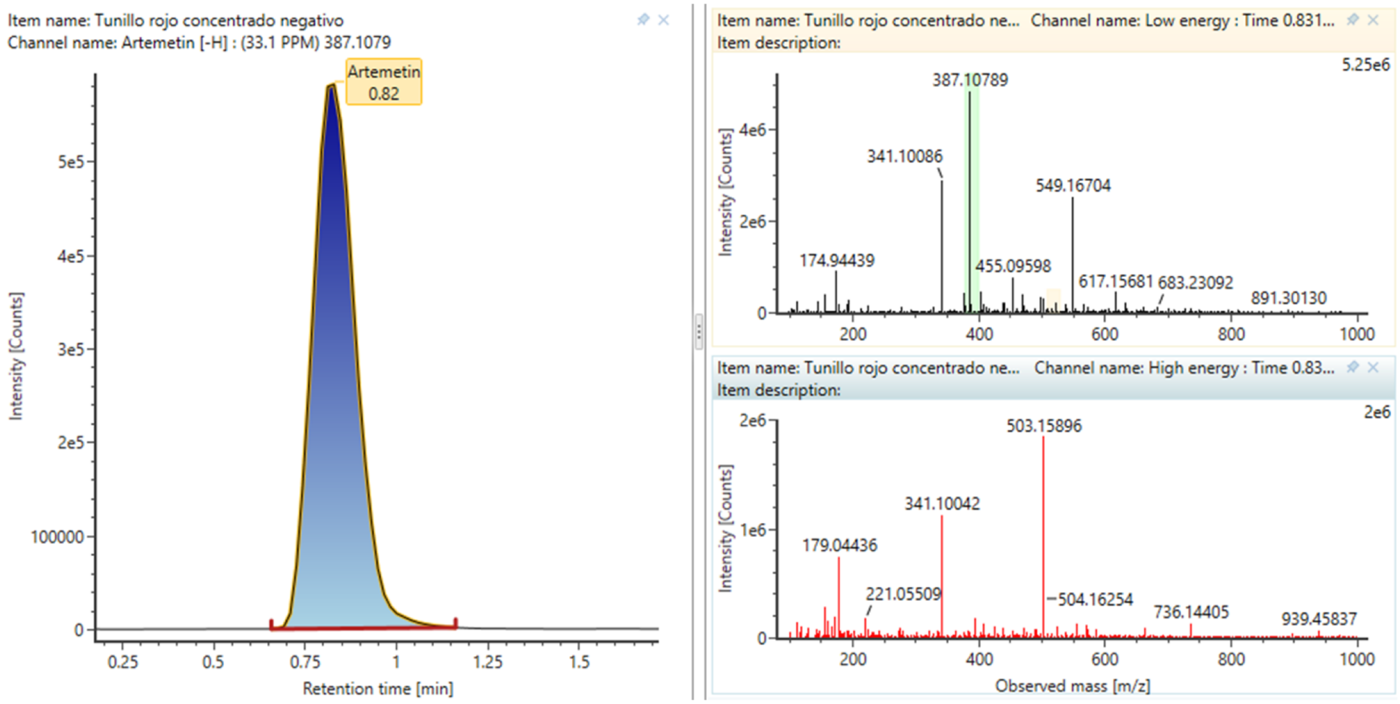

b)

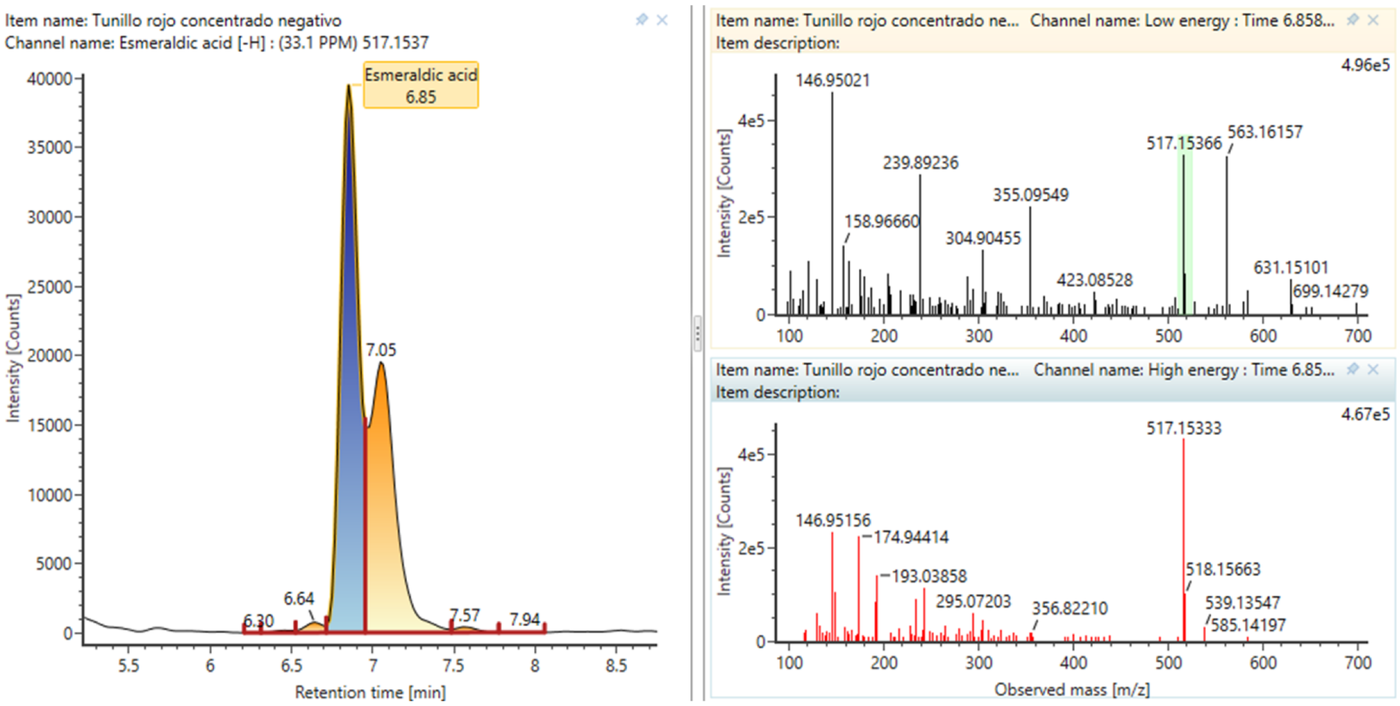

Retention times and corresponding low- and high-energy MS^E spectra of phytochemicals identified in the aqueous extract prepared from freeze-dried *Stenocereus stellatus* seeds by UPLC-QTOF-MS.

c)

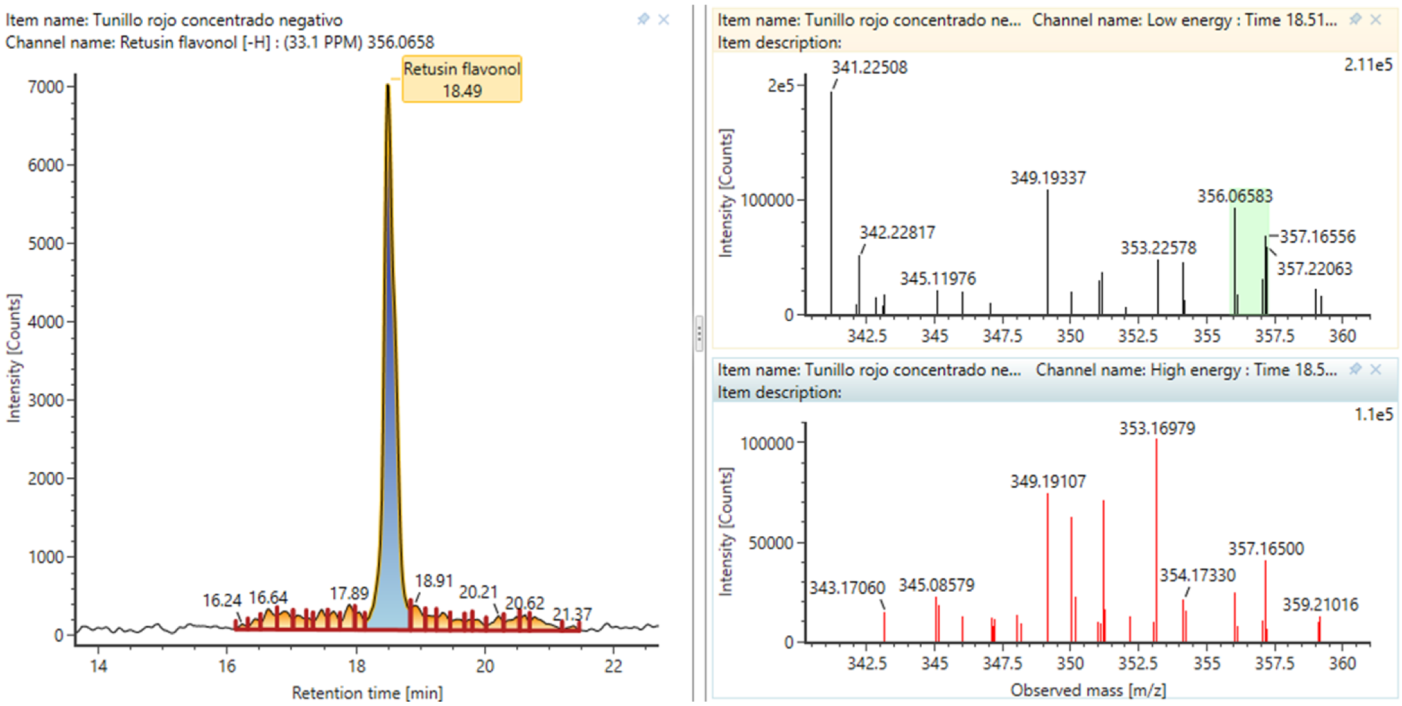

d)

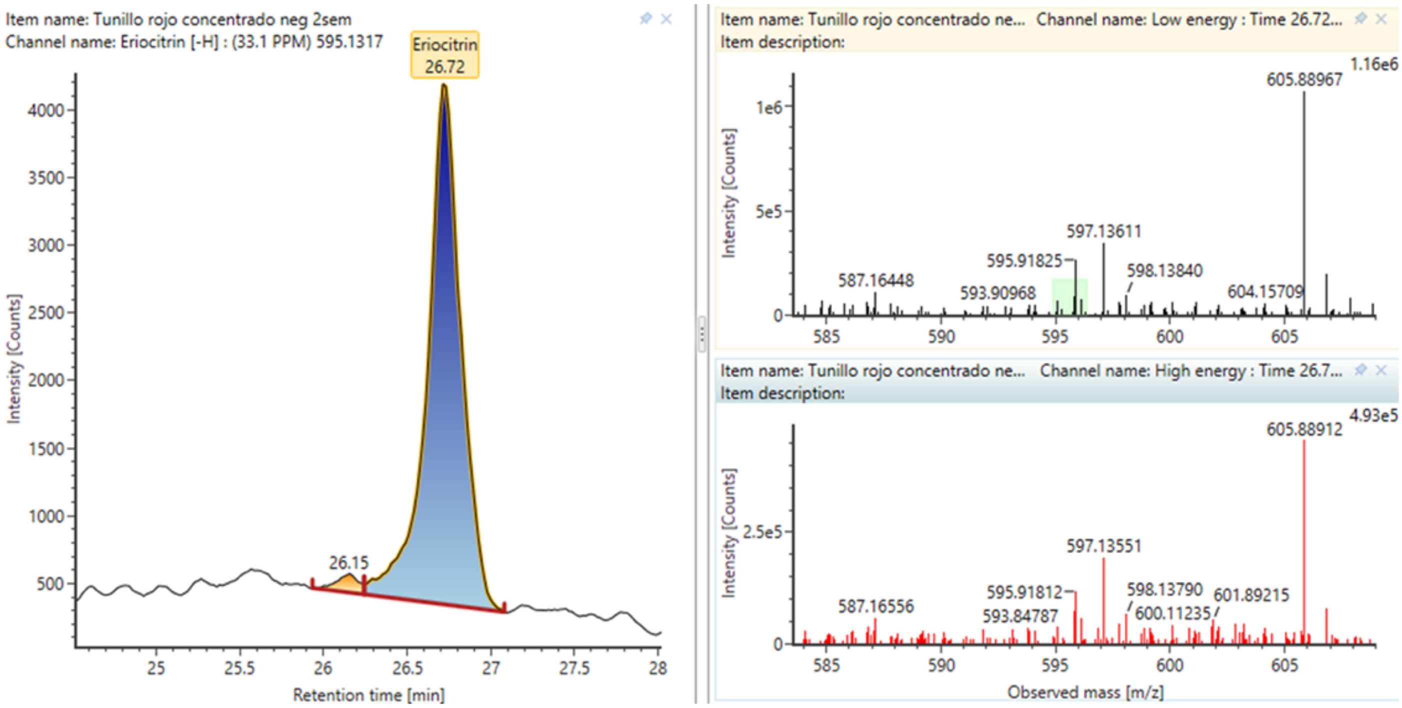

Retention times and corresponding low- and high-energy MS^E spectra of phytochemicals identified in the aqueous extract prepared from freeze-dried *Stenocereus stellatus* seeds by UPLC-QTOF-MS.

e)

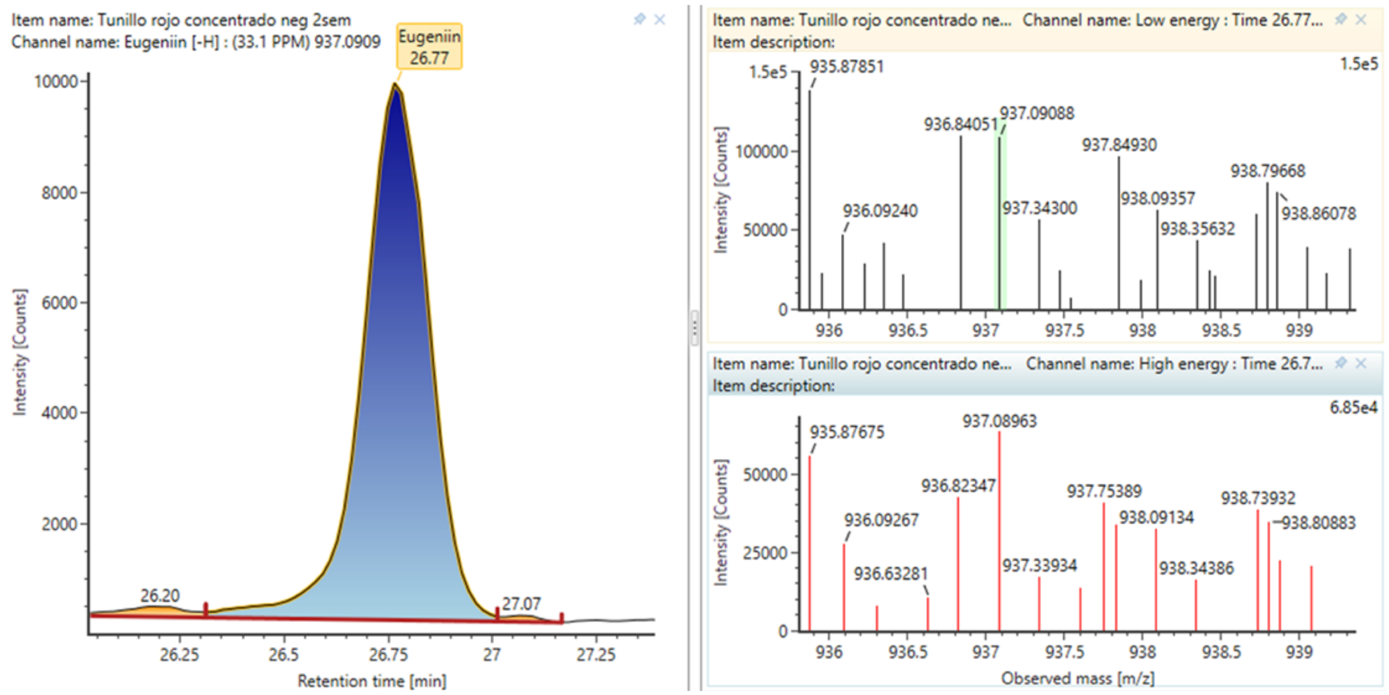

f)

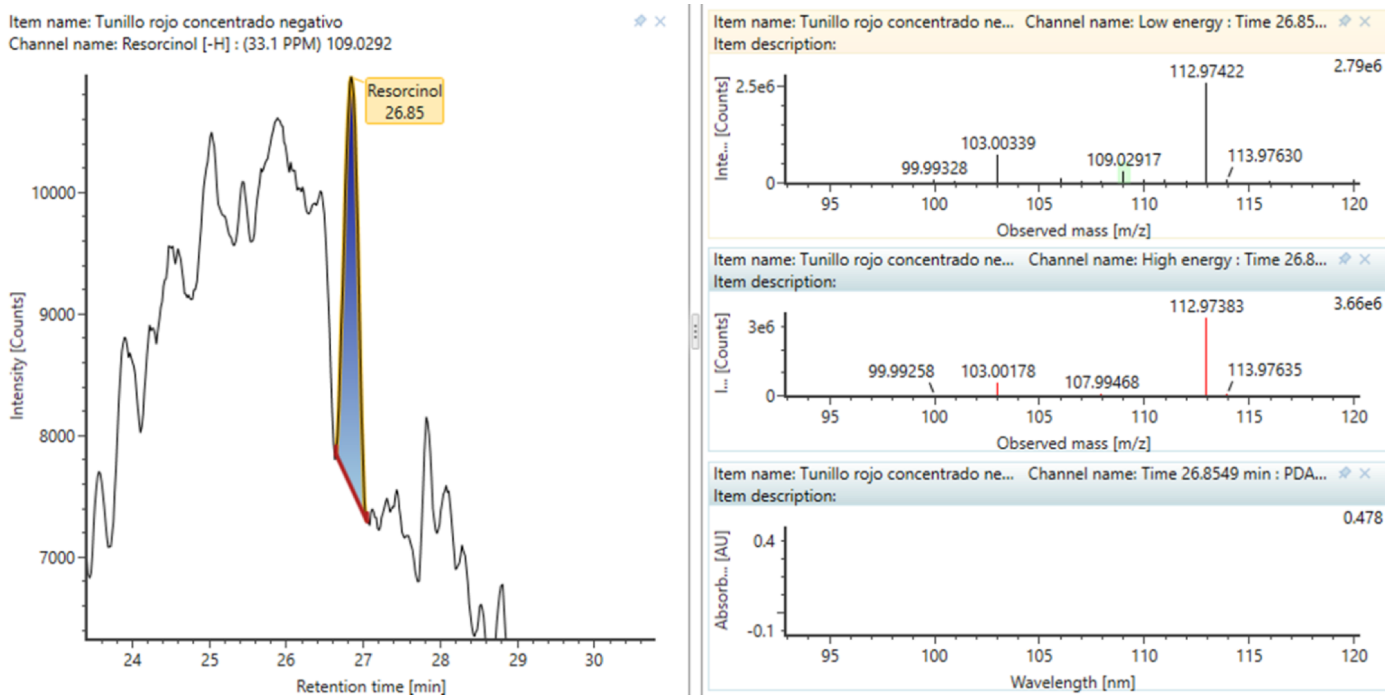

Supplement: Supplementary file 1 [file molecules-31-02964-s001.zip › Supplementary Figure S1 .pdf]
